# Elucidating the Evolutionary History of the Critically Endangered *Batagur baska* Using Complete Mitogenome

**DOI:** 10.3390/genes17101242

**Published:** 2026-10-09

**Authors:** Swati Nawani, Dhandapani Sureshgopi, Raveendran Amirtha Balan, Hemant Singh, Asim Bashir, Samrat Mondol, Abhijit Das

**Affiliations:** 1 Wildlife Institute of India, Chandrabani, Dehradun 248001, India; swatinawani327@gmail.com (S.N.); sureshgopizoo24@gmail.com (D.S.); amirthabalanrs13@gmail.com (R.A.B.); asimbashir7060@gmail.com (A.B.); 2 National Tiger Conservation Authority, B-1 Wing, 7th Floor, Pt. Deendayal Antyodaya Bhawan, CGO Complex, New Delhi 110003, India; hemantifsrj@gmail.com

**Keywords:** *Batagur baska*, estuarine turtle, mtDNA, whole-genome sequencing, endangered species, phylogeny

## Abstract

**Background/Objectives:** *Batagur baska* is a critically endangered turtle, primarily found in the estuaries and tidal regions of large rivers. *B. baska* has experienced extensive population declines across most of its range, with no confirmed wild populations remaining. This study aims to generate and characterize a complete mitogenome of *B. baska*, as well as assess its phylogeny and evolutionary history **Methods:** Eight mitochondrial genome were generated using next-generation sequencing (NGS). The dataset was used to assess phylogenetic position using Bayesian and Maximum Likelihood approaches, as well as to estimate divergence times. **Results:** The assembled mitogenomes (16,731, 16,715, 16,662, 16,654, 16,642, 16,582, 16,540, 16,527 bp) comprises 37 genes, including 13 protein-coding genes (PCGs), 22 transfer RNA genes, and two ribosomal RNA genes, along with a fully resolved non-coding control region. The genome exhibits a typical vertebrate organization with a nucleotide composition heavily biased toward A + T content, consistent with other Geoemydidae turtles. This mitochondrial genome data is combined with existing genetic information to evaluate the species’ phylogenetic position. The Bayesian and Maximum likelihood phylogenetic analysis confirmed a close genetic relationship between *B. baska* and *B. affinis*. Divergence time estimation analyses indicated that the lineage leading to *B. baska* diverged at approximately 9.12 million years ago (Ma) (95% HPD: 5.11–13.24 Ma), supporting a Miocene origin. Comparative analyses of the newly generated and previously published mitogenomes identified nucleotide variation, synonymous and nonsynonymous substitutions, and indels, present new mitogenome variation not known before. **Conclusions:** The research provides a complete mitochondrial genome for the species, offering robust phylogenetic and divergence time estimates. This mitogenomic resource also provides a baseline for future population levels which is required to guide potential breeding programs and reintroduction efforts, as well as for long-term monitoring.

## 1. Introduction

The Northern River Terrapin (*Batagur baska*) is a large, estuarine and riverine turtle [1]. Historically, it was considered a single widespread species, *B. baska* sensu lato, distributing from eastern India, Bangladesh, to the Malay Peninsula, and Indonesia encompassing populations now recognized as *B. baska* and *B. affinis* [2,3]. A comprehensive taxonomic revision divided it into two species, confining *B. baska* to Eastern India, Bangladesh, and Myanmar [3].

Once abundant, this species has now suffered extensive extirpations across most of its former range, with no confirmed wild populations documented in recent times [2,4]. In Bangladesh, limited sightings over the last two decades suggest a substantial decline in wild populations similar to other regions [1,4,5,6,7].

During the 19th century, Sundarban in India hosted a substantial population of *B. baska* but by the 1970s the population reduced drastically, with only around 10 breeding females estimated by the mid 1990s [8,9]. However, no confirmed records of wild population have been documented or reported in India since 1995 and in Bangladesh since 2007 [1,7]. The population decline is attributed to multiple factors, including mangrove loss, exploitation of eggs and adults, illegal trafficking, sand mining, and additional threats such as boat strikes, destructive fishing methods, bycatch, nesting habitat loss, and flash floods [1,7,10,11,12,13,14].

This alarming decline has earned it a place among the 35 most critically endangered turtle species globally; it has been classified as “ecologically extinct” and is listed in Appendix I of CITES [7,15]. Recognizing the urgent need of intervention, the ex situ conservation program for *B. baska* was initiated in India and Bangladesh. In India, the program initiated at Sajnekhali, Sundarban Tiger Reserve (STR) in 2012, and has 397 individuals in captivity [2].

Additionally, Madras Crocodile Bank Trust holds one male (from Vienna Zoo) and two females (rescued from a market in West Bengal) producing 64 juveniles [2]. Furthermore, Bangladesh has 565 individuals, while Myanmar has three females. An assurance colony in Austria houses three adult females, two adult males, and fifteen juveniles [2,14].

Effective conservation of critically endangered species like *B. baska* requires a multi-pronged approach including both ecological and genetic data. While a recent study has addressed ecological gaps, integrating genetic data offers a more powerful complementary perspective [2,16]. Mitogenomic data can provide crucial information on genetic diversity, population structure, demography, and adaptive variations that may not be captured through ecological studies alone [17]. Thus, it can inform conservation decisions such as population supplementation, assisted migrations and designation of management units, when interpreted with ecological data [18].

Some detailed studies of comparative mitogenomes across South and Southeast Asian freshwater turtles identified key structural, nucleotide, spacer differences that define specific lineages. For example, the complete mitogenome of the endangered Assam roofed turtle *Pangshura sylhetensis*, is 16,568 bp in size [19]. The mitogenome of its sister congener, the Indian tent turtle (*P. tentoria*), is 16,657 bp in size and possesses two copies of TTCTCTTT tandem repeat motifs in its control region [20]. In turn, the entire mitogenome of the endangered *Melanochelys tricarinata*, is 16,745 bp in size, where the control region harbors two different tandem repeat motifs, one termination-associated sequence (TAS) of TACAT, and four conserved sequence blocks, CSB-F, CSB-1, CSB-2, CSB-3 [21].

Comparative nucleotide analyses have been conducted for other freshwater turtles inhabiting the region. The crowned river turtle (*Hardella thurjii*) has a completed mitogenomic sequence of 16,699 bp in length [22]. Meanwhile, the Malaysian giant turtle (*Orlitia borneensis*) has the longest reported mitogenome of any turtle species thus far at 17,588 bp, of which the large portion of 2080 bp comprises the control region (CR) [23].

Among the turtles’ studies, the genus *Batagur* have been well-represented in mitogenome studies. The Burmese roofed turtle (*B. trivittata*) has a 16,463 bp mitogenome [24], while *B. kachuga* and *B. dhongoka* have mitogenomes of 16,155 and 15,620 bp respectively [25]. Another study on *B. kachuga* reported a mitogenome length of 16,517 bp [26]. The complete mitogenome of *B. borneoensis* is 16,397 bp in length [27]. *B. affinis affinis* and *B. affinis edwardmolli* have mitogenomes of 16,526 bp and 16,630 bp [28,29]. A recent study has examined genetic diversity and population demography of *B. baska* using mitochondrial DNA with a length 16,280 bp [30]. The differences in reported mitogenome lengths may reflect both genuine structural variation and differences in sequence recovery, assembly, or annotation among studies.

In the present study, we employed next-generation sequencing (NGS) to generate the whole mitochondrial genome of *B. baska* using two approaches. These approaches are: (a) target-enrichment strategies implemented before sequencing and (b) short read whole-genome sequencing followed by mitogenome assembly. The objectives of this study were to characterize mitochondrial genome and gene organization, reconstruct the phylogenetic relationship, and estimate divergence time to understand the evolutionary placement of the species. In addition, multiple individuals were sequenced to evaluate the structural variation within the mitogenome. While the protein coding genes rRNAs and tRNAs would presumably be highly conserved, the control region which often displays variation was of particular interest. The control region is one of the informative mitochondrial markers for population-level genetic diversity studies and can thus lay the groundwork for future assessment of genetic diversity and population structure in *B. baska*, complementing microsatellite markers and thus aiding in conservation genetic monitoring of this critically endangered species. We have also performed a comparative analysis between our mitogenomes and previously published mitogenome of *B. baska* and is further discussed in results.

## 2. Materials and Methods

### 2.1. Biological Sampling

**Figure 1:**
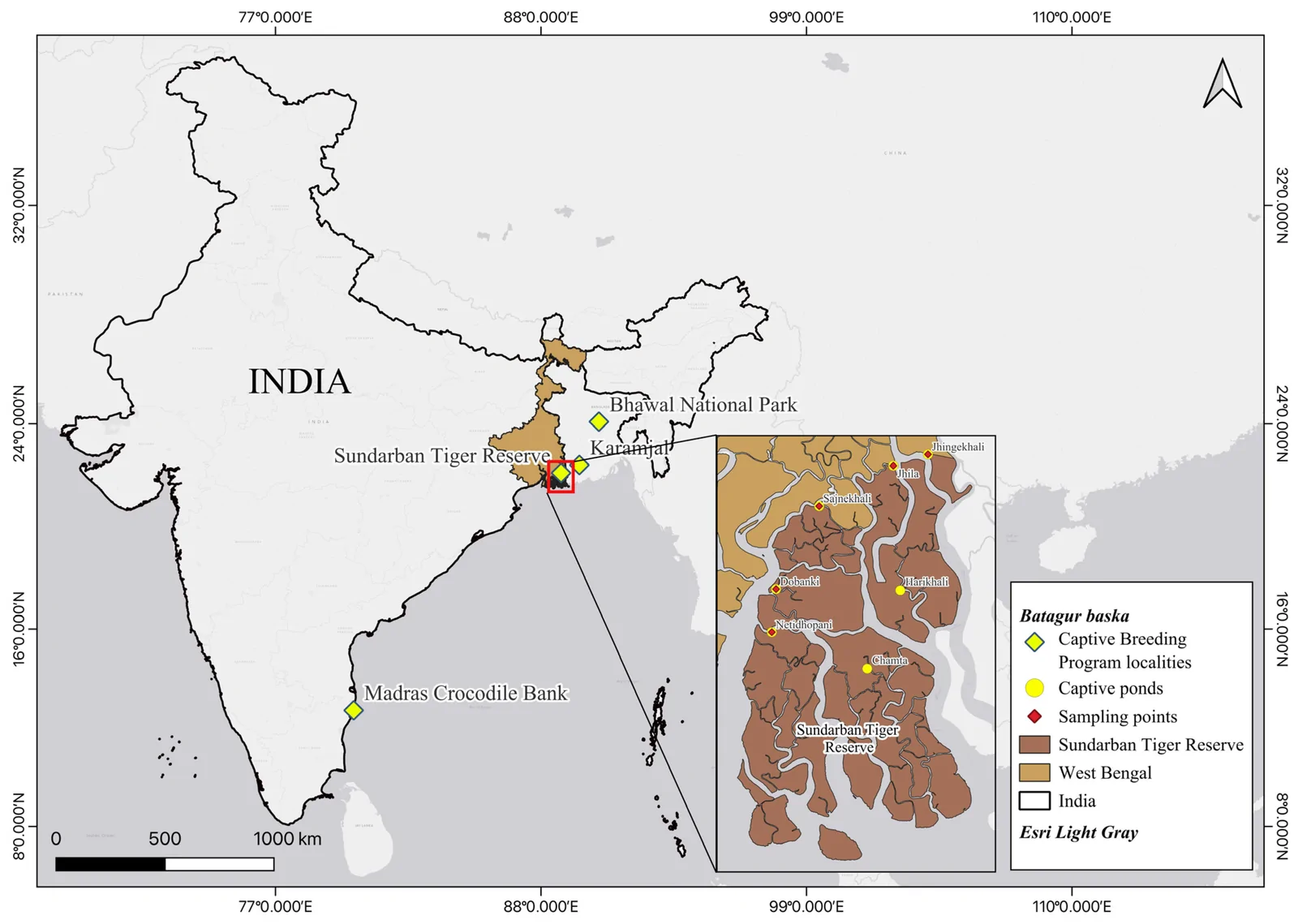
Sampling localities and captive conservation programs of *Batagur baska*. Samples were collected from five captive camps in Sundarbans, India: Jhingekhali (PZ210612), Sajnekhali (PZ210611, PZ210613), Jhila (PZ210608), Dobanki (PZ210609, PZ210610), and Netidhopani (PZ210614, PZ210615). The figure also indicates the localities of other captive conservation programs for *B. baska* in India and Bangladesh.

Biological samples were taken from an ex situ conservation facility for *B. baska*, situated in the Sundarban Tiger Reserve (STR) (Figure 1). STR is situated on the Ganges Delta, formed by the confluence of the Ganges, Brahmaputra, and Meghna rivers, at the northern boundary of the Bay of Bengal [31,32], and is one of the places where this species was historically distributed [8,9]. Approximately 40% of the Sundarbans (~4260 km^2^ area) lies within India, while the remaining (~6017 km^2^) is situated in Bangladesh [33], characterized by a closed network of rivers, channels, and creeks, leading to the formation of numerous flat islands. This area is designated as an UNESCO World Heritage Site since 1987 and a Biosphere Reserve in 1989 [34].

During January 2024, blood samples were collected from eight individuals aseptically (~2 mL) by the Forest Department veterinary officials from the subcarapacial venous for each individual. The animals were released to their respective ponds after sampling. The collected blood was then preserved in EDTA-containing vacutainers (BD Diagnostics, Franklin Lakes, NJ, USA) and transported to the laboratory at 4 °C within 24 h to Wildlife Institute of India for storage at −20 °C until further analysis.

### 2.2. DNA Extraction and Next-Generation Sequencing

Eight complete mitochondrial genomes of *B. baska* were generated using two sequencing approaches. Four of the samples (S10, S12, S20, and S40) underwent mitochondrial enrichment by differential (high-speed) centrifugation prior to next-generation sequencing at Unipath Specialty Laboratory Ltd., Ahmedabad, India whereas the other four samples (1T, 2T, 3T and 4T) were sequenced using whole-genome sequencing (Supplementary Table S1).

For the mitochondrial-enrichment approach, DNA was extracted using Alexgen Blood gDNA Kit (Alexius Biosciences, Ahmedabad, Gujarat, India) following the manufacturer’s instructions and concentrations were estimated using a Qubit 4.0 fluorometer (Invitrogen, Life Technologies, Carlsbad, CA, USA). The DNA was amplified using LCO1490/HCO2198 and 8F/1492R primer pairs and the amplified products were resolved on a 1.8% agarose gel. Sequencing libraries were prepared from 50 ng DNA using Twist NGS Library Preparation Kit and sequenced as 2 × 150 bp paired-end reads on an Illumina NovaSeq 6000 platform (Illumina, Inc., San Diego, CA, USA). The reads were quality-filtered and adapter-trimmed using Trim Galore v0.6.4_dev.

For whole-genome sequencing genomic DNA was isolated using MasterPure DNA Purification Kit (Lucigen, Middleton, WI, USA) and concentrations and purity were assessed using Qubit and NanoDrop respectively. Whole-genome shotgun libraries were prepared using NEBNext Ultra II FS DNA Library Prep kit (New England Biolabs, Inc., Ipswich, MA, USA) and sequenced as 2 × 150 bp paired-end reads on an Illumina NovaSeq 6000 (Illumina, Inc., San Diego, CA, USA) at BRIC-Institute of Life Sciences (BRIC-ILS), Bhubaneswar, India, generating approximately 28–34 Gbp of raw data per library.

### 2.3. Mitogenome Assembly, Validation and Annotation

For the four samples sequenced at Unipath, quality-filtered reads were assembled independently using NOVOPlasty v4.3.1, CLC Genomics Workbench v24, and GetOrganelle v1.7.7.0 using the default parameters [35,36,37]. CLC Genomics Workbench assemblies were selected for downstream analyses. To verify the mitochondrial origin of the assemblies, the resulting sequences were searched against the NCBI nucleotide (NT) database using BLASTN v2.13.0+ [38]. For each sample, a mitochondrial scaffold of approximately 16–16.6 Kilobase pairs (kbp) showing similarity to the complete mitochondrial genome of *Batagur kachuga* (NC_069558.1) was recovered. The selected assemblies were annotated using MITOS2 (http://mitos2.bioinf.uni-leipzig.d, 10 August 2026) [39], and the predicted open-reading frames were manually checked using the ExPASy Translate tool (http://www.expasy.org, 10 August 2026) [40].

For the four WGS-derived samples, raw reads were quality assessed using FastQC v0.12.1 and trimmed with Trimmomatic v0.39, while Cutadapt v5.2 was used to remove additional adapters when necessary [41,42,43]. Mitochondrial genomes were first reconstructed de novo from trimmed reads using GetOrganelle v1.7.7.0 in animal mitochondrial mode [37]. The assemblies used default k-mer sizes of 21, 45, 65, 85, and 105, while the extension rounds were set to 15; additional assemblies with reduced-read reduction and max-read parameters were performed for sample 1T and 4T when necessary. A high-quality, complete mitochondrial genome of *B. baska* (PZ210608) retrieved de novo was used as a reference for reference-guided assembly of the remaining WGS samples, following a de novo-to-reference-guided assembly strategy previously applied in mitochondrial-genome reconstruction [44].

In the reference-guided mitochondrial genome reconstruction, trimmed reads were mapped to the reference genome using BWA-MEM v0.7.19 [45]. The obtained alignments were processed using SAMtools v1.2, while the number of mitochondrial reads was estimated using flagstat and idxstats [46]. Variants were detected using bcftools mpileup and bcftools call, followed by the quality assessment of individual variants using the QUAL > 30 threshold and sample specific consensus sequences generation with bcftools v1.22.2 [47]. The assemblies were then evaluated using genome length, ambiguous nucleotides, read-depth distribution, and BLASTN similarity [48] to assess mitochondrial identity and examine discrepancies. The validated mitochondrial genomes were annotated using MITOS2 with the vertebrate mitochondrial genetic code [39].

### 2.4. Genome Mapping and Annotation

Initial annotation of the 13 protein-coding genes (PCGs), two ribosomal RNAs (rRNAs), and 22 transfer RNAs (tRNAs) was performed using MITOS2 [39], with gene boundaries assessed by comparison with closely related chelonian mitochondrial genomes. Gene boundaries were manually curated by confirming start and stop codons against homologous mitochondrial proteins, inspecting open reading frames for frameshifts, and adjusting feature boundaries where necessary to preserve biologically plausible coding sequences and conserved motifs. Intergenic spacers and overlapping regions were also recorded. A circular genome map of PZ210608 was generated from the curated annotation using Proksee (https://proksee.ca/, 10 August 2026). GC content and GC skew were calculated in 500-bp sliding windows advanced by 1 bp, with GC skew defined as (G − C)/(G + C), and the Proksee-derived GC-skew values were independently recalculated from the PZ210608 sequence for verification [49].

### 2.5. Identification and Secondary-Structure Analysis of Mitochondrial tRNAs

All 22 mitochondrial tRNAs were re-identified from the finalized PZ210608 sequence using tRNAscan-SE v2.0.12 in vertebrate mitochondrial search mode [50]. Predicted secondary structures were visualized with RNAplot in the ViennaRNA Package v2.7.2 [51]. Predicted base/pairs were classified as Watson–Crick, G–U wobble, or other non-Watson–Crick pairs, and extended unpaired regions were recorded. Previously noted AAU–UUA sequence features were examined at the nucleotide-pair level. The tRNA-Ser2 gene was additionally screened across 16 *Batagur* mitogenomes, comprising the eight newly generated *B. baska* sequences, two published *B. baska* sequences, and six additional *Batagur* taxa. For quantitative comparison, tRNA-Ser2 sequences from PZ210608 and six congeners were analyzed with RNAfold v2.7.2, recording sequence length, minimum free energy, predicted base pairs, unpaired nucleotides, and the longest internal unpaired region [51].

### 2.6. Nucleotide Composition and Strand Asymmetry

Nucleotide composition (A, T, G, and C counts and percentages) was calculated for complete mitogenomes and for the 13 PCGs, two rRNAs, 22 tRNAs, and control region. Comparative nucleotide composition and strand asymmetry were evaluated across 12 *Batagur* mitogenomes: eight newly generated *B. baska* mitogenomes and four closely related references, *B. affinis affinis*, *B. affinis edwardmolli*, *B. borneoensis*, and *B. kachuga* (Supplementry Table S1). AT skew and GC skew were calculated as (A − T)/(A + T) and (G − C)/(G + C), respectively [52]. Ambiguous nucleotides were excluded from nucleotide-composition estimates and alignment gaps were excluded where applicable. Complete-mitogenome values were derived from the original sequences, whereas feature-level estimates were calculated from the standardized annotation dataset.

### 2.7. Protein-Coding Gene Annotation and Codon-Usage Analyses

The 13 mitochondrial PCGs were confirmed by open-reading-frame inspection and comparison with homologous mitochondrial proteins. Start and termination codons were compared across *B. baska* and six *Batagur* references: *B. affinis affinis*, *B. affinis edwardmolli*, *B. borneoensis*, *B. dhongoka*, *B. kachuga*, and *B. trivittata* (Supplementary Table S1). Codons were interpreted using vertebrate mitochondrial (translation Table 2). Complete stop codons were recorded as annotated, whereas incomplete terminal signals were recorded as incomplete, consistent with the recognized occurrence of incomplete mitochondrial termination signals completed by polyadenylation [53].

Relative synonymous codon usage (RSCU) was calculated from the concatenated PCGs across the same seven *Batagur* taxa. Stop codons were excluded, leaving 60 sense codons. RSCU was calculated following Sharp and Li [54], where 1 indicates equal synonymous-codon usage and values > 1 or <1 indicate relative over- or under-representation. The resulting 7 × 60 matrix was subjected to Euclidean-distance hierarchical clustering using average linkage and displayed as a heatmap with codons grouped by amino acid [55,56].

### 2.8. Comparative Sequence Variation, Indel Analysis, and dN/dS

Comparative sequence variation was evaluated across the eight newly generated and two previously published *B. baska* mitogenomes (PV156654.1 and PV156655.1) (Supplementary Table S1). Genome-wide nucleotide differences were identified by pairwise sequence comparison, and coding-region variation was assessed at nucleotide, codon, and amino-acid levels. Insertions and deletions (indels) were identified from multiple-sequence alignments of the assemblies and manually checked against the final annotation; candidate indels within PCGs were evaluated to distinguish genuine sequence variation from assembly or annotation artefacts.

Synonymous (dS) and nonsynonymous (dN) substitution rates were estimated for the 13 PCGs across seven *Batagur* taxa, including PZ210608. Protein sequences were aligned with MAFFT v7.515, codon alignments were generated with PAL2NAL using vertebrate mitochondrial (translation Table 2), and pairwise dN and dS values were estimated with the Yang–Nielsen method implemented in YN00 of PAML v4.10.10 [57,58,59,60]. Each PCG yielded 21 pairwise comparisons among the seven taxa, giving 273 pairwise estimates across all 13 genes. Gene-wise ω values were calculated as mean dN divided by mean dS. These estimates were used to describe relative evolutionary constraint rather than as a formal site-specific or gene-specific positive-selection test [59,60].

### 2.9. Control-Region Organization and Tandem-Repeat Analysis

Mitochondrial control regions (CRs) were examined in the eight newly generated *B. baska* mitogenomes, two published *B. baska* sequences, and six comparative *Batagur* reference sequences (Supplementary Table S1). The eight newly generated CRs were extracted and aligned with MAFFT [57]. A TAS-associated motif and the conserved sequence blocks CSB-F, CSB-1, CSB-2, and CSB-3 were identified by comparative inspection following published descriptions of turtle mitochondrial CR organization [61,62]. Tandem repeats in the eight newly generated CRs were identified with Tandem Repeats Finder (TRF) using parameters 2, 7, 7, 80, 10, 50, and 2000 [63]. All TRF predictions were retained in a single supplementary worksheet, while representative long-period arrays were displayed in the main figure.

### 2.10. Mitogenome Phylogenetic Analysis

Phylogenetic relationships among *B. baska* and related Geoemydidae and Testudinidae turtles were inferred from complete mitochondrial genomes. The principal phylogenetic dataset comprised 104 complete mitochondrial genomes, including eight newly generated *B. baska* mitogenomes and publicly available sequences retrieved from GenBank (Supplementary Table S1). The sequences were aligned using MAFFT [57] and manually inspected to identify and remove ambiguously aligned or poorly aligned regions, resulting in a final alignment of 15,355 bp. Maximum-likelihood inference was performed in IQ-TREE 2 v2.4.0 [64], with ModelFinder selecting GTR + F + R5 under the Bayesian Information Criterion [65]. Branch support was assessed using 1000 ultrafast bootstrap (UFBoot) and SH-aLRT replicates [66]. *Manouria emys* (NC_007693.1) was used as the designated outgroup; *Aldabrachelys gigantea* and *Chelonoidis* spp. remained within the analyzed tree. Bayesian inference was performed in MrBayes v3.2 [67] using the 103-taxon dataset and 15,355-bp alignment, without partitioning, under GTR with gamma-distributed among-site rate variation. Two independent runs of four chains were run for 5,000,000 generations, sampling every 1000 generations, with diagnostics evaluated every 5000 generations and the first 25% discarded as burn-in. Convergence was evaluated using split-frequency standard deviation, potential scale reduction factors, and effective sample sizes; posterior trees were summarized as an all-compatible consensus and visualized in iTOL v6 [68].

A sensitivity ML analysis excluded all eight newly generated *B. baska* mitogenomes, leaving 96 reference mitogenomes under the same 15,355-bp alignment framework and GTR + F + R5 model. The same 1000 UFBoot and SH-aLRT replicates were applied. Unlike the principal figure, the sensitivity tree was fully expanded and all 96 reference sequences were shown individually. The purpose of this analysis was to test whether the broader *Batagur* relationships depended on inclusion of the newly generated assemblies.

### 2.11. Molecular Clock Divergence-Time Estimation

Divergence-time estimation was conducted in BEAST v2.7.8 [69] under a Bayesian relaxed-clock framework. Mitochondrial sequences were organized by gene or structural region, aligned with MAFFT, and manually inspected before dating. Protein-coding regions were partitioned by codon position, while 12S rRNA, 16S rRNA, and tRNAs were treated as separate partitions. Partition-specific substitution models were selected under BIC using Partition Finder 2 [70]. An uncorrelated lognormal relaxed clock and a Yule tree prior were used [71,72]. Three crown-node minimum-age constraints were implemented as lognormal priors, with hard minimum offsets of 47.0 million years ago (Ma) for Geoemydidae, 50.3 Ma for Testudinidae, and 53.0 Ma for Testudinoidea [73,74,75]. MCMC chains were run for 1 × 10^8^ generations, parameters and trees were logged at fixed intervals, and convergence and mixing were evaluated in Tracer using trace inspection and effective sample sizes. After burn-in removal, posterior trees were summarized as a maximum clade credibility chronogram with TreeAnnotator v2.7.8.

## 3. Results

### 3.1. Mitochondrial Genome Organization and Nucleotide Composition

#### 3.1.1. Genome Organization and Annotation

**Figure 2:**
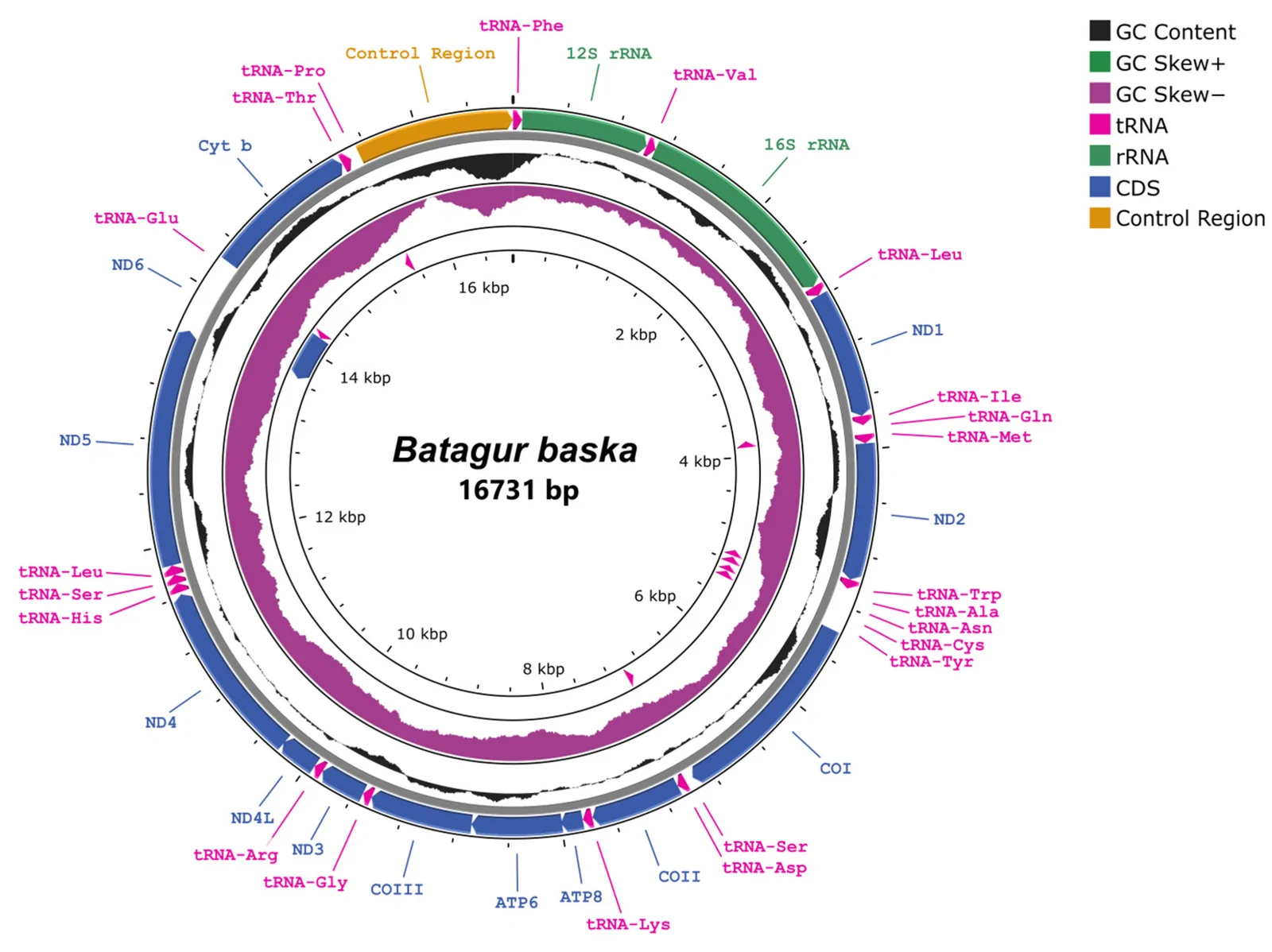
Circular mitochondrial genome map of *B. baska* PZ210608. Protein-coding genes, tRNA genes, rRNA genes, and the control region are shown with their genomic positions and orientations. Inner rings display GC content and GC skew; genome coordinates are shown in kbp.

A total of eight mitochondrial genomes were generated. The complete mitochondrial genome of *B. baska* PZ210608 is a circular molecule of 16,731 bp and comprises 13 protein-coding genes (PCGs), 22 tRNA genes, two rRNA genes, and a control region (Figure 2). Gene order and strand orientation follow the typical vertebrate mitochondrial arrangement, with no gene rearrangements or duplications detected. Detailed feature coordinates, lengths, strand assignments, and intergenic spacers or overlaps are provided in Table 1.

The curated annotation was used to generate the circular genome map and to verify gene boundaries (Figure 2). Start and stop codons were checked against homologous mitochondrial proteins, open reading frames were inspected for frameshifts, and feature boundaries were adjusted where required to retain biologically plausible coding sequences. The final annotation therefore provides the reference framework used in all downstream comparative analyses.

#### 3.1.2. Comparative Nucleotide Composition and Strand Asymmetry

**Figure 3:**
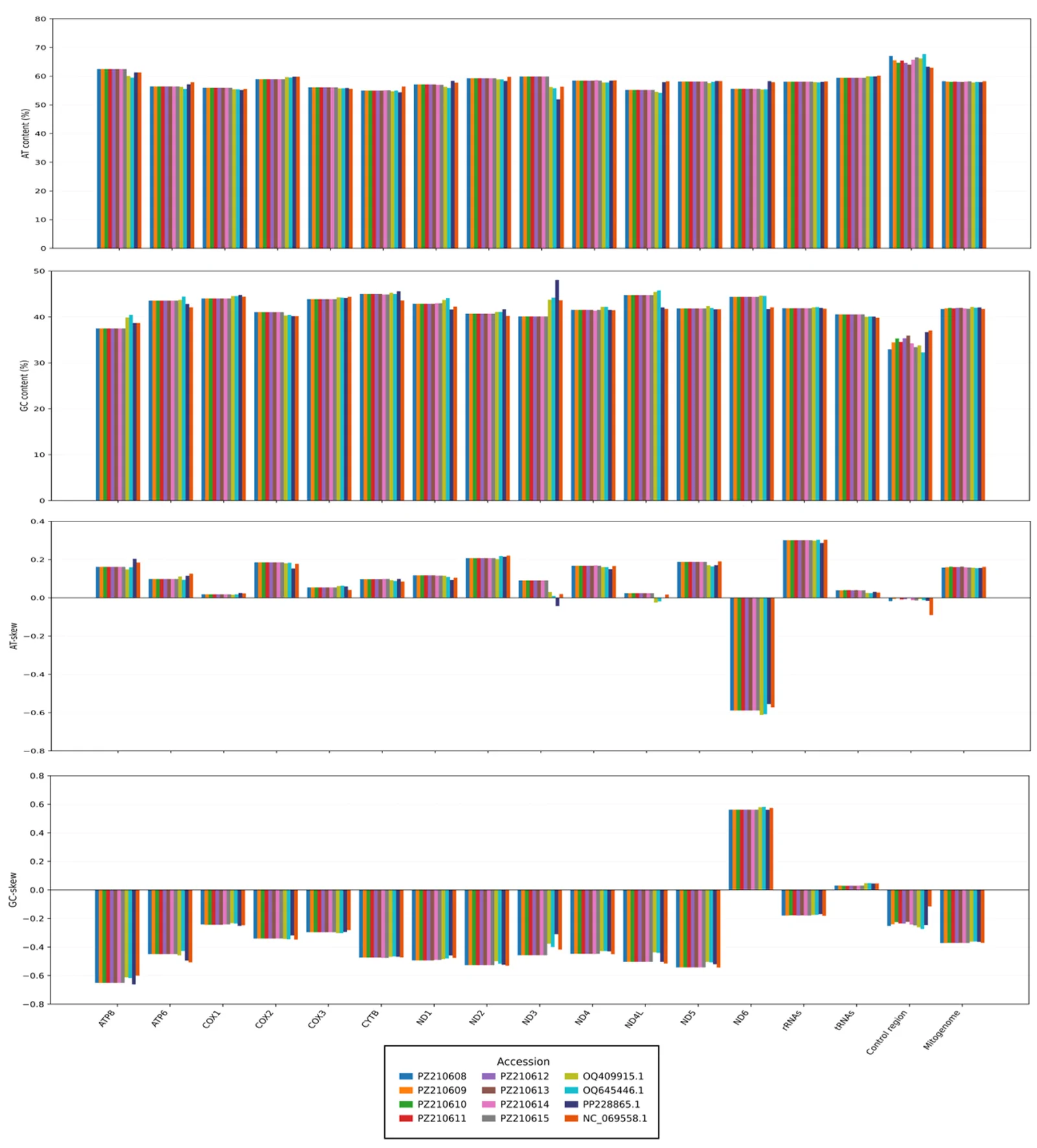
Comparative nucleotide composition and strand asymmetry of *Batagur* mitogenomes. AT content, GC content, AT skew, and GC skew are compared across the 13 mitochondrial PCGs, two rRNAs, 22 tRNAs, the control region, and complete mitogenomes for eight newly generated *B. baska* mitogenomes and four closely related *Batagur* reference mitogenomes.

Across the eight newly generated *B. baska* mitogenomes, whole-mitogenome AT content ranged from 57.99% to 58.26%, while AT skew was positive (0.158–0.163) and GC skew was negative (−0.373 to −0.371) in all eight genomes. Most PCGs showed the same positive AT-skew and negative GC-skew pattern, whereas ND6 consistently showed the opposite pattern. The control region was more AT-rich than the complete mitogenome, with AT content ranging from 64.05% to 67.06%, and displayed comparatively weak AT skew and consistently negative GC skew (Figure 3).

For PZ210608 specifically, GC skew calculated across 500-bp sliding windows advanced by 1 bp ranged from −0.687 to +0.011, with a mean of −0.371 and a median of −0.386, while GC content ranged from approximately 25.8% to 48.5%. Independent recalculation reproduced the Proksee-derived skew values. The pronounced negative GC-skew profile indicates strong strand-specific nucleotide compositional asymmetry. Such asymmetry has been associated with replication-related mutational processes in vertebrate mitochondrial genomes, but transcription and other molecular factors may also contribute. The present data are therefore interpreted as evidence of strand compositional asymmetry rather than direct evidence of a specific replication mechanism.

### 3.2. Mitochondrial rRNA and tRNA Structure

#### 3.2.1. rRNA/tRNA Organization

The PZ210608 mitogenome contains two mitochondrial rRNAs, rrnS and rrnL, and 22 tRNA genes. The rRNAs occur in the conserved positions between tRNA-Phe and tRNA-Val and between tRNA-Val and tRNA-Leu, respectively. The 22 tRNAs occupy both mitochondrial strands and together retain the characteristic vertebrate mitochondrial gene complement. Detailed gene coordinates and strand assignments are provided in Table 1.

#### 3.2.2. tRNA Secondary Structure and Conserved tRNA-Ser2 Features

**Figure 4:**
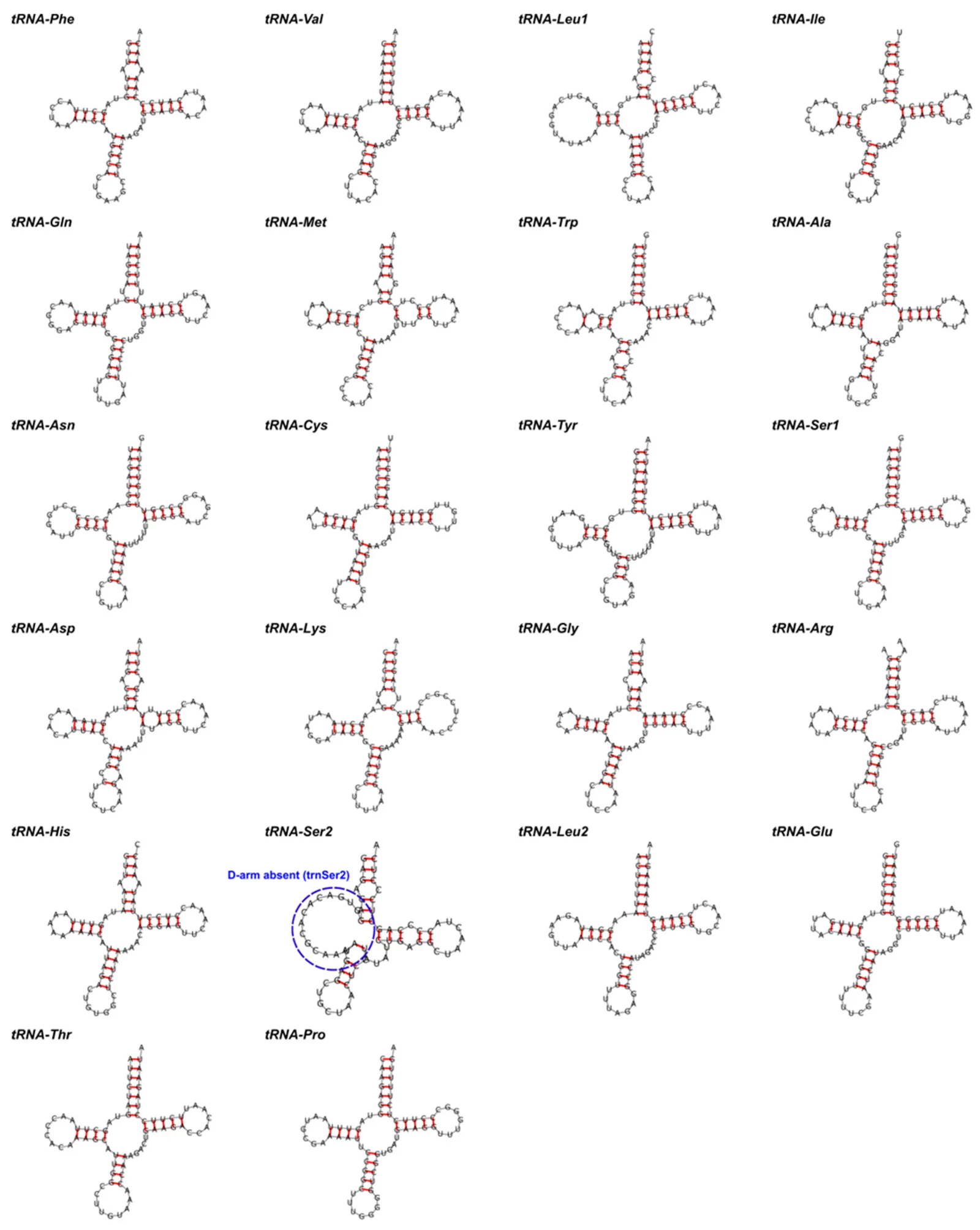
Predicted secondary structures of the 22 mitochondrial tRNAs of *B. baska* PZ210608. The marked structure highlights the D-arm-lacking architecture of tRNA-Ser2 predicted by tRNAscan-SE v2.0.12. Detailed G–U wobble-pair positions and additional structural features are provided in Supplementary Table S9. Comparative tRNA-Ser2 structures across 16 *Batagur* mitogenomes are provided in Supplementary Figure S1.

A total of 426 predicted base pairs were identified, comprising 388 Watson–Crick pairs and 38 G–U wobble pairs distributed among 11 tRNAs; no additional non-Watson–Crick pairing class was detected (Figure 4). The most conspicuous feature was the reduced architecture of mitochondrial tRNA-Ser2 (trnS2), which was predicted to lack a D-arm (Figure 4). Screening of 16 *Batagur* mitogenomes showed the same predicted D-arm absence in all sampled sequences, indicating that the feature is not restricted to PZ210608. (Supplementary Figure S1 and Figure 4) three AAT-TTA sequence features corresponding to previously noted AAU-UUA observations occurred in trnV, trnL1, and trnT; in each case, the nucleotides formed canonical A-U pairs and did not represent mismatches or additional non-canonical interactions.

Despite the conserved D-arm-lacking architecture, the residual tRNA-Ser2 structure varied among taxa. RNAfold predicted 14 paired positions and 41 unpaired nucleotides for PZ210608, whereas the six comparison taxa showed 15–27 paired positions and 17–37 unpaired nucleotides. *B. kachuga* showed a similarly reduced pairing pattern with 15 predicted base pairs, whereas *B. affinis affinis*, *B. affinis edwardmolli*, and *B. borneoensis* each showed 27 predicted base pairs (Supplementary Table S2). The predominance of canonical pairing and G–U wobble pairs indicates broadly conserved mitochondrial tRNA structure, while the repeated D-arm reduction in tRNA-Ser2 indicates a conserved architectural feature within the sampled *Batagur* lineages.

### 3.3. PCG Codon Usage and Sequence Variation

#### 3.3.1. Start- and Termination-Codon Usage

Start-codon usage was highly conserved across the seven examined *Batagur* mitogenomes. ATP8, ATP6, COX2, COX3, ND1, ND2, ND3, ND4L, ND5, ND6, and CYTB began with ATG in all taxa, whereas COX1 consistently used GTG. The only additional deviation was observed in ND4 of *B. kachuga*, which also began with GTG. Thus, the conserved initiation pattern was retained across 90 of the 91 gene-by-taxon comparisons.

Termination patterns showed greater variation. ATP8, ATP6, COX2, and ND4L consistently ended with TAA, whereas COX1 ended with AGG across all seven taxa. COX3, ND1, ND2, and ND3 consistently showed incomplete terminal signals. Variation was concentrated in ND4, ND5, ND6, and CYTB (Supplementary Table S3).

Overall, initiation-codon usage was highly conserved among the examined *Batagur* mitogenomes, whereas termination-codon composition showed modest gene- and taxon-specific variation. Under the vertebrate mitochondrial genetic code, AGA and AGG function as termination codons, consistent with mitochondrial termination mechanisms.

#### 3.3.2. Relative Synonymous Codon Usage

**Figure 5:**
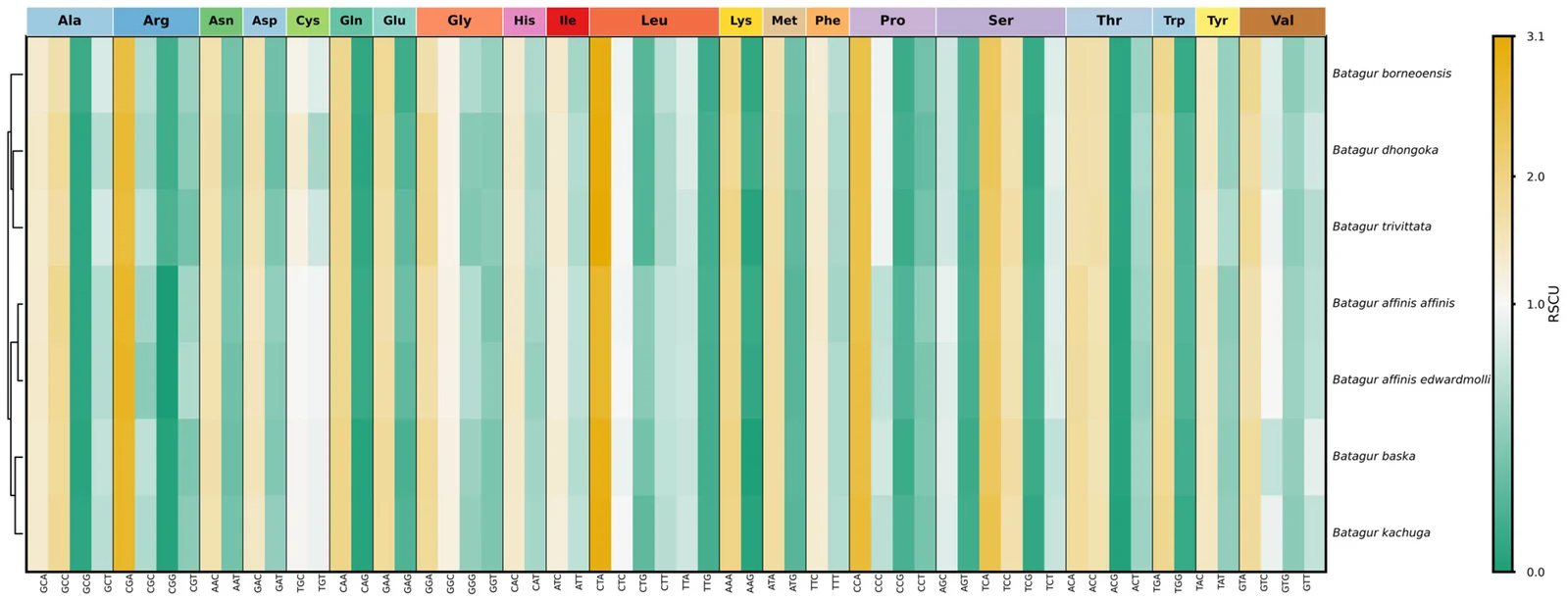
Comparative relative synonymous codon usage (RSCU) among seven *Batagur* taxa. Codons are grouped by amino acid, and taxa are clustered using Euclidean distances based on RSCU profiles. Values of 1 indicate equal synonymous codon usage; values below and above 1 indicate relative under- and over-representation, respectively. The highest RSCU is observed for CTA (Leu) in *B. trivittata* (3.09).

RSCU profiles were broadly conserved across the examined *Batagur* taxa, although pronounced preferences were observed for several synonymous codons (Figure 5). The highest RSCU was recorded for CTA (Leu) in *B. trivittata* (3.0903), followed by consistently high CTA usage across the other taxa. CGA (Arg), CCA (Pro), and TCA (Ser) also showed elevated RSCU values, whereas ACG (Thr) and GCG (Ala) were strongly underrepresented. CGG (Arg) was absent in *B. affinis affinis* and *B. affinis edwardmolli*. Thus, differences among taxa were primarily quantitative, with the direction of codon preferences remaining broadly conserved.

The conserved RSCU profiles indicate a broadly shared pattern of synonymous codon usage among the examined *Batagur* mitogenomes. Such patterns may reflect nucleotide-compositional constraints and other evolutionary forces; however, RSCU alone cannot distinguish mutational effects from selection. The codon-usage patterns are therefore treated as descriptive evidence of codon-use bias and interpreted together with nucleotide composition and dN/dS analyses.

#### 3.3.3. Comparative Nucleotide, Codon, and Amino-Acid Variation

Comparative analyses of the eight newly assembled and two previously published *B. baska* mitogenomes identified sequence variation while retaining the conserved mitochondrial gene organization. Pairwise comparison between the published BB02 mitogenome (PV156655.1) and refined PZ210608 identified 70 nucleotide substitutions, comprising 46 substitutions within PCGs and 24 substitutions within non-coding regions (Supplementary Table S4). Among the coding-region substitutions, 44 variable codons were identified, including 38 synonymous and six nonsynonymous substitutions (Supplementary Table S5). Across all ten available *B. baska* mitogenomes, 50 variable codons were identified, comprising 39 synonymous and 11 nonsynonymous variants (Supplementary Table S6), and translation identified 11 amino-acid variants distributed among COX1, ND1, ND4, ND5, and CYTB (Supplementary Table S7).

**Figure 6:**
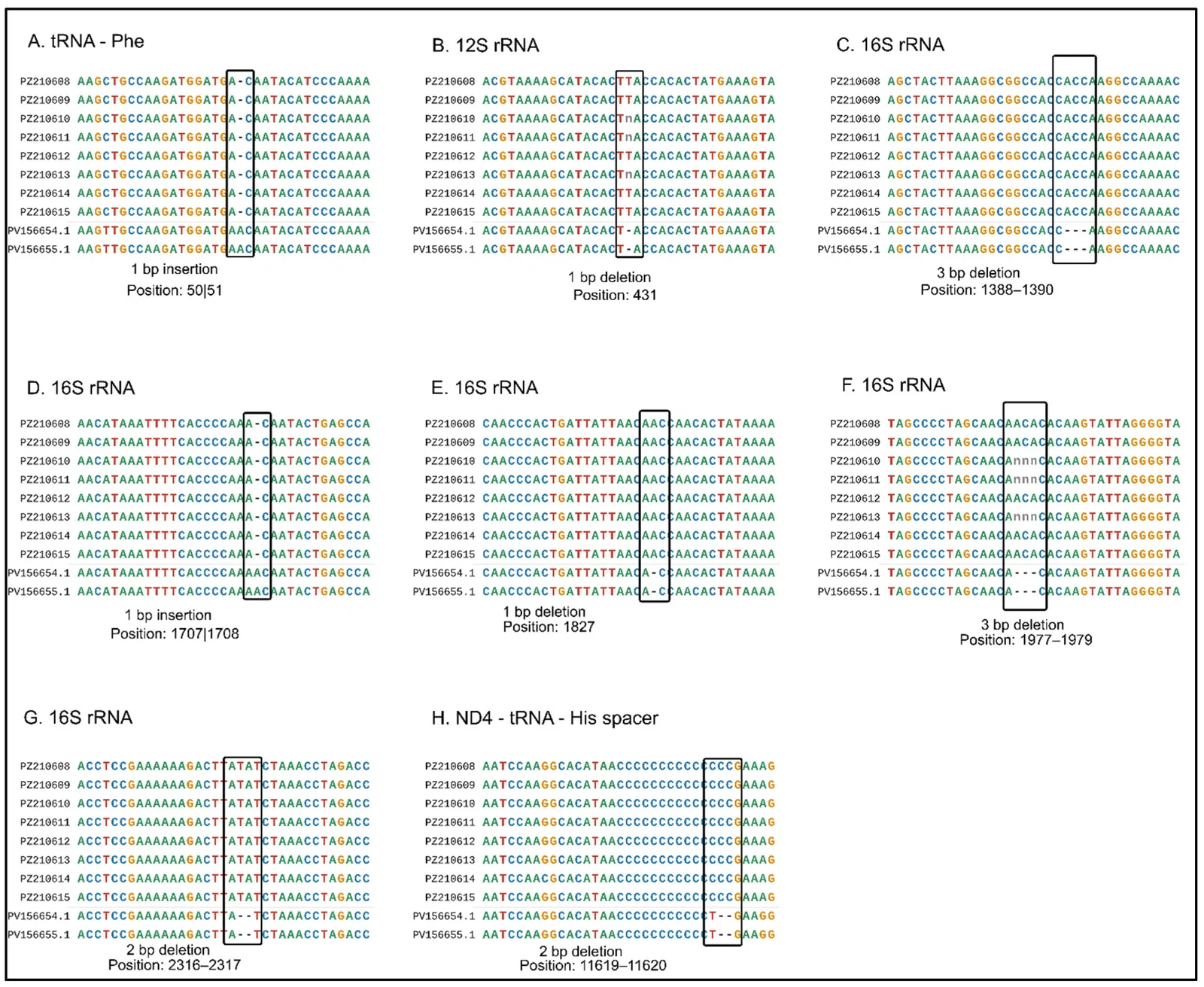
Multiple-sequence alignment showing validated insertion/deletion (indel) polymorphisms among the newly generated *B. baska* mitochondrial genomes (GenBank accession numbers PZ210608–PZ210615) and the previously published mitogenomes (PV156654.1 and PV156655.1). Indels were identified in tRNA-Phe (**A**), 12S rRNA (**B**), 16S rRNA (**C**–**G**), and the ND4–tRNA-His intergenic spacer (**H**). Boxed regions indicate the indel positions. Nucleotide bases are color-coded by identity (A, C, G, and T).

Multiple-sequence alignment identified eight indel loci, comprising one in tRNA-Phe, one in the 12S rRNA, five in the 16S rRNA, and one in the ND4-tRNA-His intergenic spacer. These indels ranged from 1 to 3 bp (Supplementary Table S8), and no validated indels were retained within the 13 PCGs after genome curation. Together, the nucleotide, codon, amino-acid, and indel comparisons provide complementary evidence that the finalized PZ210608 assembly is consistent with the variation observed across independently generated and previously published *B. baska* mitogenomes (Figure 6).

#### 3.3.4. Synonymous and Nonsynonymous Substitution Patterns

**Figure 7:**
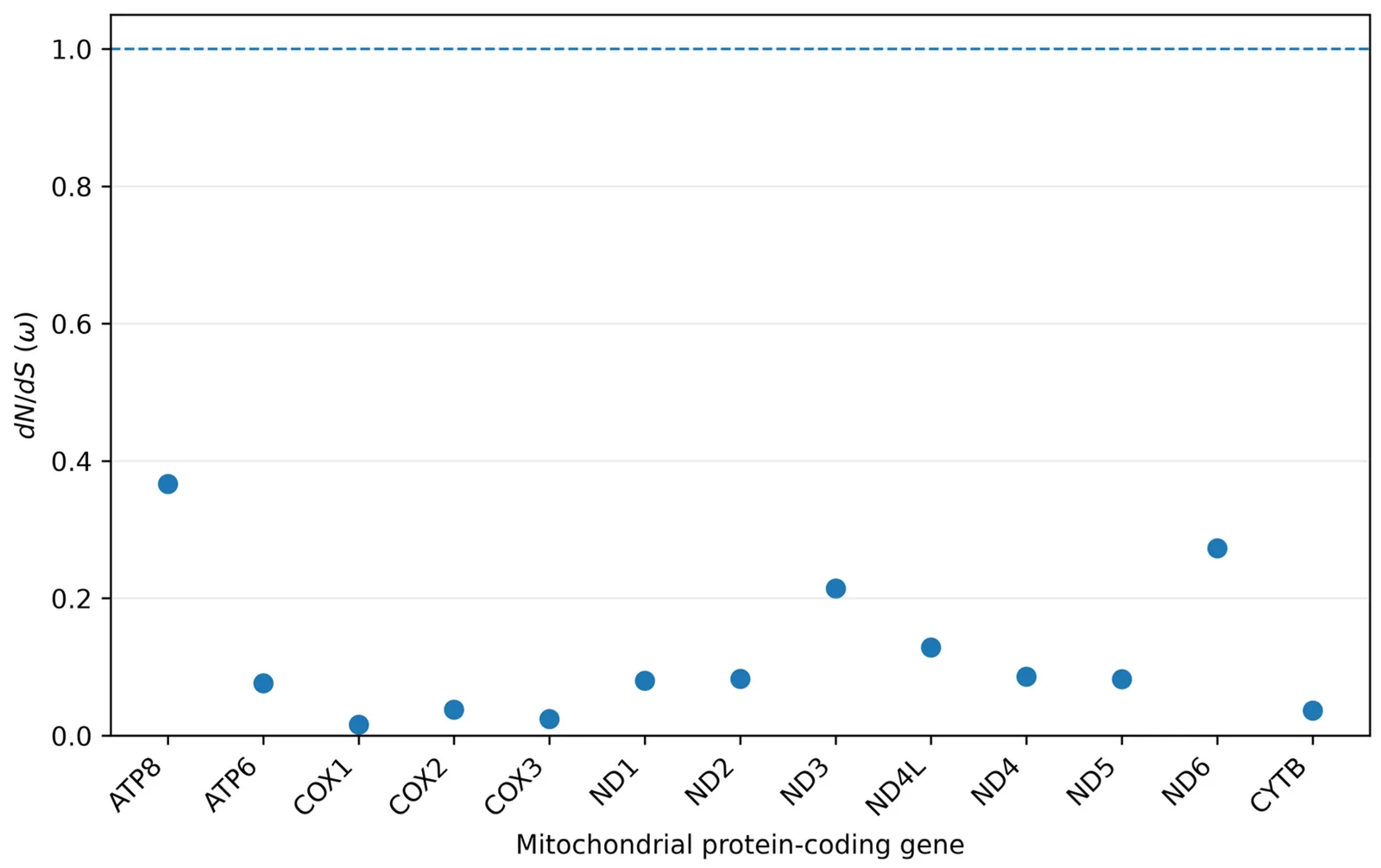
Gene-wise dN/dS ratios among seven *Batagur* taxa. Points represent gene-wise ω values calculated as mean nonsynonymous (dN) divided by mean synonymous (dS) substitution rates from 21 pairwise Yang–Nielsen comparisons for each mitochondrial protein-coding gene. The horizontal dashed line indicates ω = 1. Values below 1 are consistent with predominant purifying selection. The figure is intended to accompany the Section 3.3.4 paragraph above; the full numerical estimates are provided in Supplementary Table S9.

Gene-wise omega values were below 1 for all 13 mitochondrial PCGs, indicating predominant purifying constraint. COX1 showed the lowest omega (0.0161), followed by COX3 (0.0241), CYTB (0.0366), and COX2 (0.0379), whereas ATP8 (0.3667), ND6 (0.2730), and ND3 (0.2143) showed comparatively higher values (Figure 7). One ATP8 pairwise comparison yielded an omega of 1.1546; however, the gene-wise estimate remained below 1 and the isolated pairwise value is not interpreted as evidence of positive selection (Supplementary Table S9).

The dN/dS result therefore indicates heterogeneity in the strength of evolutionary constraint among mitochondrial PCGs, with cytochrome c-oxidase genes being more conserved than ATP8, ND6, and ND3. Because the analysis is based on pairwise YN00 estimates and gene-wise averages, it should be interpreted as evidence for relative differences in evolutionary constraint rather than as a formal test of site-specific or gene-specific positive selection.

### 3.4. Control-Region Organization and Tandem-Repeat Variation

**Figure 8:**
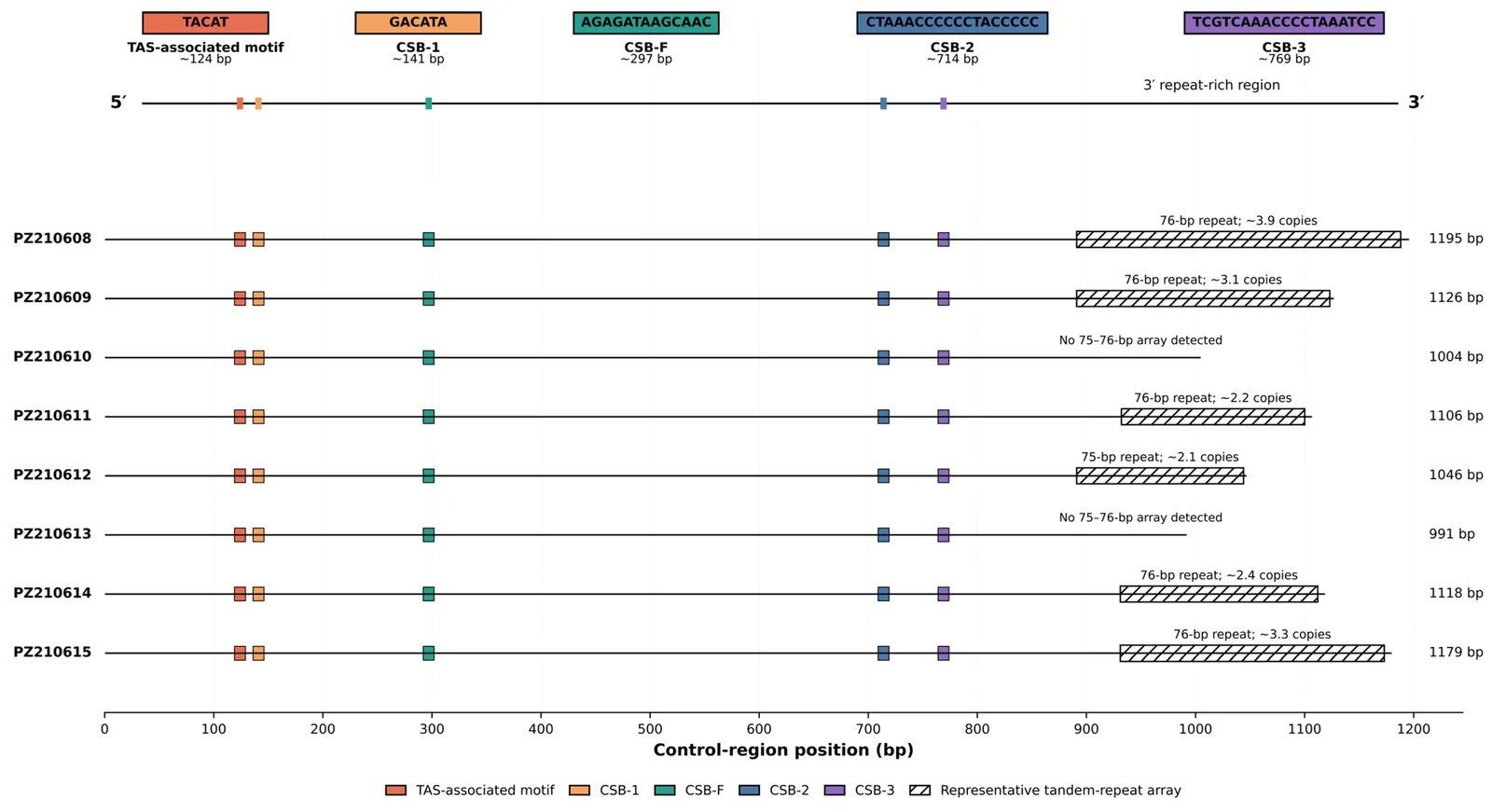
Comparative organization of the mitochondrial control region in eight newly generated *B. baska* mitogenomes.

The eight newly generated *B. baska* control regions ranged from 991 to 1195 bp, corresponding to a 204-bp difference in length. Despite this variation, all eight retained the same major organizational framework, with the TAS-associated motif and the conserved sequence blocks CSB-1, CSB-F, CSB-2, and CSB-3 occurring at approximately 124, 141, 297, 714, and 769 bp, respectively (Figure 8; Table 2). The two published *B. baska* sequences (PV156654.1 and PV156655.1) were shorter, at 754 bp, and retained the upstream conserved elements; CSB-3 could not be assessed because these sequences terminated before that region. The CSB-F sequence was identical among the eight newly generated *B. baska* mitogenomes (AGAGATAAGCAAC), whereas several comparative *Batagur* sequences contained the closely related AGAAATAAGCAAC variant.

Across the eight newly generated control regions, 30 tandem-repeat predictions were identified (Supplementary Table S10). A 75–76-bp tandem-repeat array was present in six genomes—PZ210608, PZ210609, PZ210611, PZ210612, PZ210614, and PZ210615—with approximately 2.1–3.9 copies represented in Figure 7. PZ210610 and PZ210613 lacked this 75–76-bp array but contained shorter-period repeat profiles. The repeat predictions were concentrated mainly in the 3′ portion of the control region, consistent with the repeat-rich organization reported in geoemydid turtles. Overall, the conserved positions of the major control-region elements across genomes spanning a 204-bp length range indicate a relatively stable organizational framework. Variation in tandem-repeat architecture, including the presence or absence of the 75–76-bp array and differences in repeat-period profiles, may contribute to inter-genome variation in control-region length. Thus, the observed variation appears to be localized primarily within the repeat-rich portion of an otherwise conserved control-region structure.

### 3.5. Mitogenome Phylogeny

#### 3.5.1. Principal Maximum-Likelihood and Bayesian Inference

**Figure 9:**
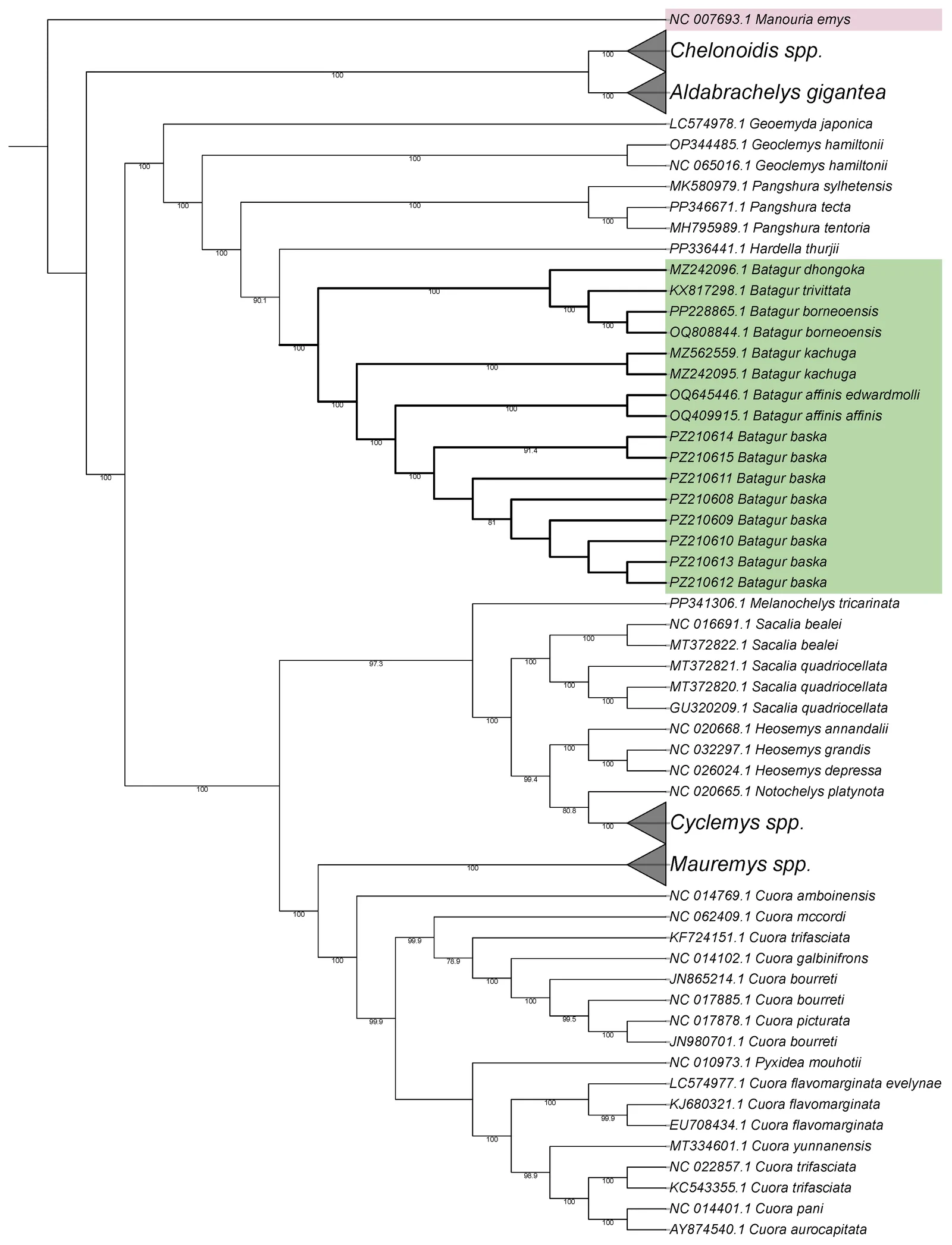
Principal maximum-likelihood phylogeny inferred from complete mitochondrial genomes of *B. baska* and related turtles. The analysis included 104 mitogenomes with a final trimmed alignment of 15,355 bp. Node labels indicate ultrafast bootstrap (UFBoot) support from 1000 replicates, with values ≥ 50% shown. *Batagur* sequences are highlighted in green, and *Manouria emys* was designated as the outgroup. Grey triangles indicate selected clades collapsed for graphical clarity; taxa and accession numbers represented within the collapsed clades are listed in Supplementary Table S1.

The maximum-likelihood analysis recovered a strongly supported clade comprising all eight newly generated *B. baska* mitogenomes (UFBoot = 100), with short internal branches indicating limited mitochondrial differentiation among the sampled individuals. The newly generated sequences formed a distinct *B. baska* lineage relative to the other sampled *Batagur* sequences. The Bayesian analysis recovered the same placement with maximal posterior probability (PP = 1.00), and its overall topology was broadly congruent with the ML reconstruction (Figure 9; Supplementary Figure S2).

These results support the distinct mitochondrial lineage of the sampled *B. baska* individuals within *Batagur*. However, the mitochondrial topology represents a maternal genealogy and should not be interpreted as a complete species-tree reconstruction. The strong support for the sampled *B. baska* clade indicates consistent mitochondrial assignment of these individuals but does not resolve nuclear population structure or historical introgression.

#### 3.5.2. 96-Taxon Sensitivity Analysis

A complementary maximum-likelihood sensitivity analysis excluding the eight newly generated *B. baska* mitogenomes recovered the same major *Batagur* relationships as the principal analysis. The reference sequences of *B. borneoensis*, *B. kachuga*, and *B. affinis* formed strongly supported groupings, while *B. trivittata* and *B. dhongoka* retained their respective positions within *Batagur*. Overall, the major *Batagur* topology remained unchanged in the absence of the newly generated assemblies, indicating that their inclusion did not materially influence the broader mitochondrial phylogenetic relationships (Supplementary Figure S3).

### 3.6. Molecular Clock Divergence-Time Analysis

**Figure 10:**
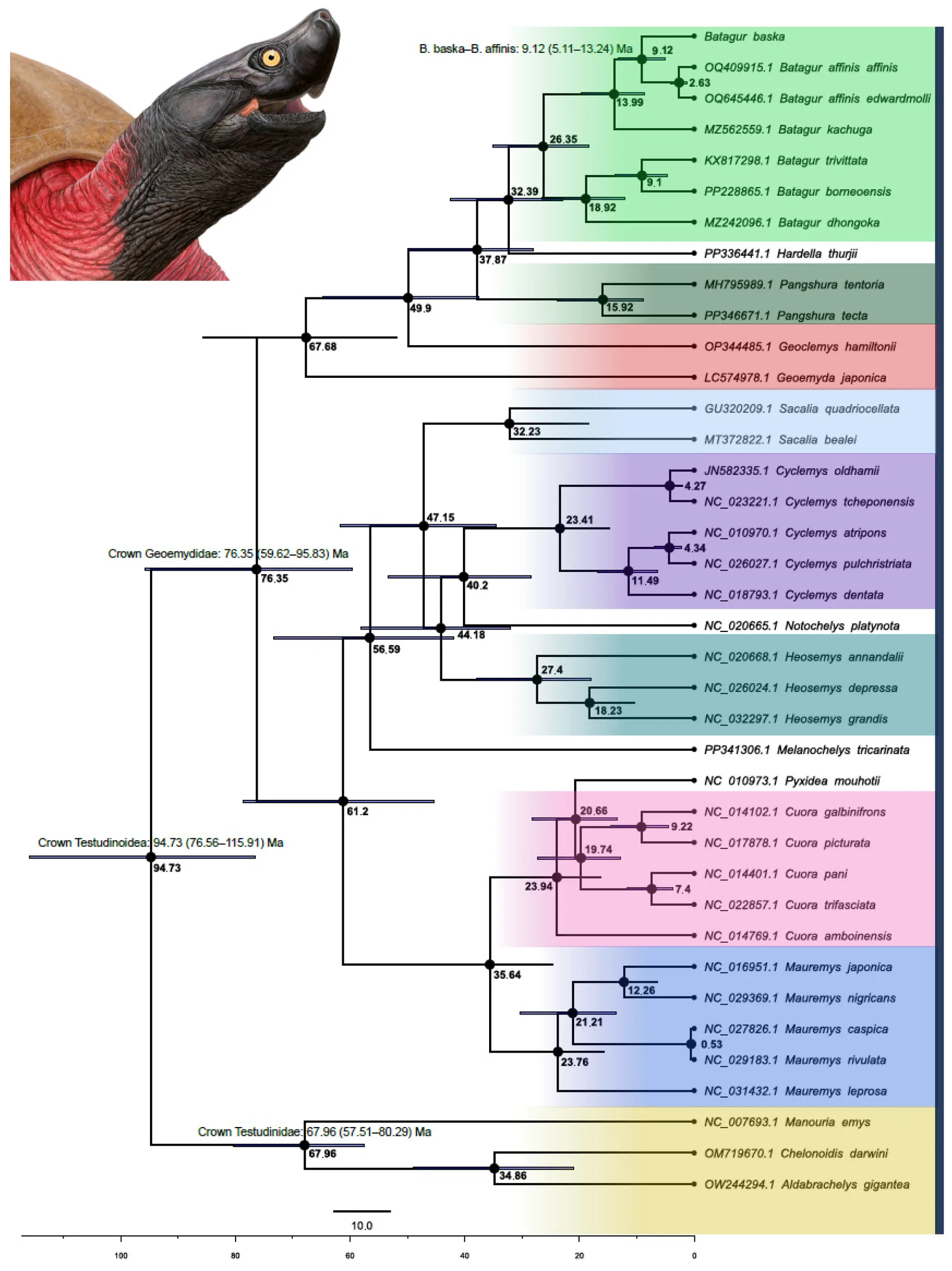
Bayesian time-calibrated phylogeny of Testudinoidea showing posterior divergence-time estimates and 95% highest posterior density intervals. The estimate for the lineage leading to *B. baska* is 9.12 Ma (95% HPD: 5.11–13.24 Ma).

The MCC chronogram estimated the crown age of Testudinoidea at 94.73 Ma (95% HPD: 76.56–115.91 Ma), crown Geoemydidae at 76.35 Ma (95% HPD: 59.62–95.83 Ma), and crown Testudinidae at 67.96 Ma (95% HPD: 57.51–80.29 Ma). At the focal scale, the divergence involving the lineage leading to *B. baska* was estimated at 9.12 Ma (95% HPD: 5.11–13.24 Ma), placing the divergence in the Miocene. Because the interval spans several million years, this estimate is best treated as a probabilistic Miocene-scale estimate rather than a precise point date (Figure 10).

## 4. Discussion

The complete mitochondrial genome of *B. baska* was successfully sequenced and annotated, giving detailed insight into structure and composition of mitogenome. The length of 16,731 bp is closer to the mitogenome sizes of the closely related *B. affinis affinis* (16,625 bp) and *B. affinis edwardmolli* (16,630 bp). The mitogenome retains the canonical vertebrate mitochondrial gene complement and organization, while the comparative analyses demonstrate that this organization is highly conserved across the newly generated *B. baska* sequences. The broad predominance of Watson–Crick pairing, together with a limited number of G–U wobble pairs, indicates that the mitochondrial tRNA repertoire of *B. baska* retains a stable secondary-structure framework. The consistent absence of the D-arm in tRNA-Ser2 across all 16 sampled *Batagur* mitogenomes is notable because it suggests that this structural reduction is conserved rather than being a sequence-specific feature. A similar reduction in the secondary structure of a mitochondrial serine tRNA has been reported in the geoemydid *Pangshura tentoria*, where in tRNA-Ser1 lacks the conventional DHU arm [20]. D-arm loss is also a well-documented feature of metazoan mitochondrial tRNAs, particularly tRNA-Ser [76]. Variation in the residual tRNA-Ser2 stem-loop organization nonetheless indicates that structural details can differ among lineages even when the major architectural reduction is conserved.

One of the major strengths of this study is the establishment of a comprehensive mitochondrial genomic baseline with a complete control region, a marker important for population genetic studies due to its high variability [77]. The control-region comparison indicates that the major conserved sequence elements occupy similar relative positions across the newly generated *B. baska* genomes despite a 204-bp range in total CR length. The greater variability in the repeat-rich three-prime region, together with the occurrence of 75–76-bp arrays in six of eight genomes and shorter-period repeats in the remaining two, suggests that repeat architecture contributes to CR length variation. The evidence supports a conserved organizational framework with localized structural variability rather than wholesale reorganization of the control region, consistent with patterns documented in turtle mitochondrial CRs [20,61,62]. This is particularly relevant given the findings of [30], who identified extremely low nucleotide diversity (π = 0.0012) and only five haplotypes within the captive population at the Sundarbans Tiger Reserve. These findings are consistent with previous microsatellite analyses that found high relatedness among the founder captive populations in Bangladesh and Austria, and a severe historical bottleneck and high relatedness [78]. In addition, CR of mitogenome is highly variable and contains large amount of A-T content. The CR sequences possess a variety of repetitive elements that differ in length and are formed by particular motifs that create hairpin loops [79]. It is postulated that such structures are formed due to specific signals required to terminate mitochondrial DNA replication [22]. The CR can be influenced by various mechanisms, including the rearrangement of genes that arise from different origins of replication, dimeric mitogenomes, and gene loss that occurs randomly or non-randomly. These processes impact the structural diversity of the mitochondrial genome, thereby helping understand the evolutionary mechanisms of mitochondrial DNA [62,80].

Mitogenome-based phylogenetic reconstruction produced consistent placement of the newly generated *B. baska* sequences across both ML-based and Bayesian analysis. These analyses support monophyly of the lineages, provide strong evidence for species identification and indicate stable phylogenetic placement within *Batagur*. This clear separation of *B. baska* from the other *Batagur* species supports its taxonomic status and underscores its conservation value as a genetically distinct lineage. The present findings are in agreement with earlier mitochondrial studies on Geoemydidae turtles, in which strong phylogenetic resolution was achieved using complete mitogenomes [81,82].

The time-calibrated analysis also provides an intriguing insight into evolutionary history of *B. baska*. According to the relaxed-clock chronogram, the divergence epoch involving the lineage leading to *B. baska* occurred during the Miocene (mean 9.12 Ma; 95% HPD 5.11–13.24 Ma). This suggests that *B. baska* represents a lineage with substantial evolutionary depth rather than a very recent derivative within *Batagur*. This temporal placement provides a testable hypothesis for future work integrating paleodrainage and climatic history of South and Southeast Asia with explicit biogeographic modeling and multilocus demographic inference. At the same time, the credible interval spans several million years and molecular dating is sensitive to calibration choices and prior specification; accordingly, the divergence estimate is best interpreted probabilistically and at the Miocene scale, rather than as a precise point date.

The divergence time analysis has located the split of the lineage leading to the present-day *B. baska* and its sister taxon, *B. affinis*, in the Late Miocene at 9.12 Ma (95% HPD: 5.11–13.24 Ma). The placement of the split within the Late Miocene gives an approximate date when the two sister clades ceased to be a single population. Whereas the distribution of *B. baska* is currently confined to the Ganges–Brahmaputra delta and Sundarbans of South Asia, *B. affinis* is found in Southeast Asia inhabiting rivers and estuaries of the region (4).

The Late Miocene has been recognized as a period of significant geological and climatic changes in South and Southeast Asia. The Neogene tectonic evolution of the Himalayan–Tibet region has influenced patterns of both Himalayan erosion and Asian paleoclimate [83]. Meanwhile, the Cenozoic tectonic activity has modified the paleogeography of Southeast Asia multiple times [84]. These processes are likely to have played a role in shaping riverine and coastal habitats and biogeographic connections across the region.

The current disjunction in the distribution of *B. baska* and *B. affinis* between South and Southeast Asian drainage basins may well have a geological origin. However, the divergence time analysis does not directly show what particular geoclimatic change may have caused the split between the two lineages. The Miocene tectonic and climatic events in South and Southeast Asia should thus be considered as providing a possible explanation for the vicariance of the sister clades but not a certain cause of their differentiation. The present dataset does not include explicit ancestral-area reconstruction, paleodrainage modeling, or multilocus demographic inference. Accordingly, the most defensible interpretation is that the Miocene age provides a temporal hypothesis that can be evaluated against future reconstructions of South and Southeast Asian drainage history, climatic change, and coastal-estuarine connectivity. The broad HPD interval and the known sensitivity of molecular dating to calibration choice and prior specification also argue against over-interpretation of the point estimate.

The establishment of this complete reference genome is timely since the species is now regarded as ecologically extinct in the wild and there have been no confirmed sightings of it in recent times within its historical range. The results significantly improve the recent genomic data for the species. A similar study has reported mitogenome of only 16,280 bp in *B. baska*, attributed to difficulty of assembling the control region [30]. The difference in length, approximately 450 bp, can be attributed to distinct sequencing strategies and sampling limits. They have used a non-invasive sampling method (mucus swabs and feces), which is important for low disturbance but often yields DNA of variable quality and quantity. They employed a sanger sequencing approach and noted inherent problems in alignment of the hypervariable, repetitive motifs of the control region. Our study was able to successfully resolve the repetitive and AT-rich motifs of the control region.

Comparison of the newly assembled and previously published *B. baska* mitogenomes revealed nucleotide differences while confirming that mitochondrial genome organization and gene content remain highly conserved. Whereas 70 nucleotide substitutions were detected, most coding regions variants were synonymous, and only a few resulted in amino acid substitutions. The predominance of synonymous changes is consistent with the strong evolutionary constraints acting on vertebrate mitochondrial protein-coding genes, where mutations that alter protein function are less likely to be retained.

The eight independently assembled mitogenomes served to further reassess sequence variation reported in earlier *B. baska* mitochondrial genomes. By corroborating the general nucleotide states of conservation seen across individual genomes, the results provide additional support for the data reported here.

In addition, all verified insertion/deletion polymorphisms were confined to RNA genes and a non-coding intergenic region, whereas protein-coding genes were free of confirmed indels after genome curation. Evaluating variation at the nucleotide, codon, amino acid, and indel levels allowed sequence differences to be interpreted in a broader genomic context rather than from a single line of evidence. This integrated approach strengthens confidence in the mitochondrial genome assembly and provides a useful reference for future studies of the evolutionary history, phylogeography, and conservation genetics of *B. baska*.

## 5. Conclusions

The integrated analyses establish a complete and curated mitochondrial reference for *B. baska* and show that its genome retains the conserved organization typical of vertebrate mitochondrial DNA. Comparative analyses reveal stable nucleotide composition, conserved tRNA architecture with repeated D-arm reduction in tRNA-Ser2, highly conserved initiation-codon usage, non-random but broadly shared synonymous codon preferences, and predominant purifying constraint across mitochondrial PCGs. The control region retains a conserved sequence-element framework while showing repeat-associated length variation. The concordant ML and BI analyses support a well-defined mitochondrial *B. baska* lineage, and the relaxed-clock analysis places the lineage leading to *B. baska* in the Miocene, although the broad uncertainty interval warrants cautious interpretation. The resulting mitogenomic resource provides a foundation for future phylogeographic, demographic, and conservation-genetic studies, particularly when integrated with nuclear genomic and ecological data.

## 6. Limitations and Future Perspectives

The complete mitogenomes generated in this study provide valuable insights; however, several limitations should be acknowledged. Mitochondrial DNA is a single, non-recombining, maternally inherited marker, and so phylogenetic relationships and divergence patterns inferred here may reflect maternal lineage history rather than complete evolutionary trajectories of species. Future studies would benefit from the inclusion of nuclear markers or genome-wide data. It will provide a more comprehensive and biparentally inherited perspective on the population structure, genetic diversity and evolutionary history of *B. baska*. Additionally, broader sampling across the captive ponds and other extant population, would better capture genetic diversity and demographic pattern and help inform future conservation practices.

## Figures and Tables

**Table 1:** Organization of the mitochondrial genome and characteristics of annotated genes. Start and End indicate the nucleotide positions of each gene in the mitochondrial genome. bp denotes base pairs. Strand indicates the orientation of each gene, with “+” representing the heavy (H) strand and “−” representing the light (L) strand. Size (bp) represents the length of each gene in base pairs. Anticodon indicates the anticodon sequence of the corresponding transfer RNA (tRNA) gene; “—” indicates not applicable because the gene is not a tRNA. Intergenic nucleotides (bp) represent the number of nucleotides between the end of a given gene and the start of the following gene. A value of 0 indicates that adjacent genes are contiguous, whereas a negative value indicates an overlap between adjacent genes; the absolute value represents the number of overlapping nucleotides. For example, an intergenic value of −1 indicates that the two adjacent genes overlap by one nucleotide.

Gene	Start	End	Strand	Size (bp)	Anticodon	Intergenic Nucleotides (bp)
trnF	1	69	+	69	GAA	0
rrnS	70	1034	+	965	—	0
trnV	1035	1104	+	70	TAC	0
rrnL	1105	2705	+	1601	—	0
trnL2	2706	2781	+	76	TAA	0
nad1	2782	3749	+	968	—	0
trnI	3750	3820	+	71	GAT	−1
trnQ	3820	3890	−	71	TTG	−1
trnM	3890	3958	+	69	CAT	0
nad2	3959	4997	+	1039	—	0
trnW	4998	5068	+	71	TCA	1
trnA	5070	5138	−	69	TGC	12
trnN	5151	5223	−	73	GTT	29
trnC	5253	5318	−	66	GCA	6
trnY	5325	5395	−	71	GTA	1
cox1	5397	6947	+	1551	—	−12
trnS2	6936	7006	−	71	TGA	0
trnD	7007	7076	+	70	GTC	0
cox2	7077	7763	+	687	—	1
trnK	7765	7837	+	73	TTT	1
atp8	7839	8006	+	168	—	−10
atp6	7997	8680	+	684	—	−1
cox3	8680	9463	+	784	—	0
trnG	9464	9531	+	68	TCC	1
nad3	9533	9881	+	349	—	0
trnR	9882	9951	+	70	TCG	0
nad4l	9952	10,248	+	297	—	−7
nad4	10,242	11,618	+	1377	—	18
trnH	11,637	11,705	+	69	GTG	0
trnS1	11,706	11,774	+	69	GCT	−1
trnL1	11,774	11,846	+	73	TAG	0
nad5	11,847	13,655	+	1809	—	−5
nad6	13,651	14,175	−	525	—	0
trnE	14,176	14,243	−	68	TTC	5
Cytb	14,249	15,388	+	1140	—	4
trnT	15,393	15,465	+	73	TGT	0
trnP	15,466	15,536	−	71	TGG	0
Control Region	15,537	16,731	+	1195	—	—

**Table 2:** Comparative characterization of mitochondrial control regions across 16 comparative accessions, including conserved-element positions and control-region availability. Entries are one-based start positions within the extracted control-region sequence.

Taxon	Accession	Dataset Role	CR Availability	Extracted CR Sequence Length (bp)	TAS-Associated	CSB-1	CSB-F	CSB-2	CSB-3
*B. baska*	PZ210608	Newly generated	Complete	1195	124 bp	141 bp	297 bp	714 bp	769 bp
*B. baska*	PZ210609	Newly generated	Complete	1126	124 bp	141 bp	297 bp	714 bp	769 bp
*B. baska*	PZ210610	Newly generated	Complete	1004	124 bp	141 bp	297 bp	714 bp	769 bp
*B. baska*	PZ210611	Newly generated	Complete	1106	124 bp	141 bp	297 bp	714 bp	769 bp
*B. baska*	PZ210612	Newly generated	Complete	1046	124 bp	141 bp	297 bp	714 bp	769 bp
*B. baska*	PZ210613	Newly generated	Complete	991	124 bp	141 bp	297 bp	714 bp	769 bp
*B. baska*	PZ210614	Newly generated	Complete	1118	124 bp	141 bp	297 bp	714 bp	769 bp
*B. baska*	PZ210615	Newly generated	Complete	1179	124 bp	141 bp	297 bp	714 bp	769 bp
*B. baska*	PV156654.1 (BB01)	Published	Truncated	754	124 bp	141 bp	297 bp	714 bp	Not assessable
*B. baska*	PV156655.1 (BB02)	Published	Truncated	754	124 bp	141 bp	297 bp	714 bp	Not assessable
*B. affinis affinis*	OQ409915.1	Reference	Available	1084	138 bp	199 bp	274 bp (variant)	691 bp	745 bp
*B. affinis edwardmolli*	OQ645446.1	Reference	Available	1109	65 bp	214 bp	289 bp (variant)	706 bp	760 bp
*B. trivittata*	NC032300.1	Reference	Available	947	117 bp	214 bp	289 bp (variant)	707 bp	761 bp
*B. borneoensis*	PP228865.1	Reference	Partial	883	117 bp	214 bp	289 bp (variant)	706 bp	760 bp
*B. kachuga*	NC069558.1	Reference	Partial	653	117 bp	216 bp	291 bp (variant)	Not assessable	Not assessable
*B. dhongoka*	NC069559.1	Reference	Very partial	106	Not assessable	Not assessable	Not assessable	Not assessable	Not assessable

Note. Conserved-element entries are one-based start positions (bp) within the respective extracted control-region sequence. TAS-associated motif = TACAT; CSB-1 = GACATA; CSB-F = AGAGATAAGCAAC in *B. baska*, whereas AGAAATAAGCAAC represents the CSB-F variant detected in several comparative *Batagur* sequences. “Not assessable” indicates that the available control-region sequence was insufficient to evaluate the corresponding element.

## Data Availability

The sequences have been submitted to GenBank, with accession numbers PZ210608–PZ210615.

## Supplementary Materials

The following supporting information can be downloaded at: https://www.mdpi.com/article/10.3390/genes17101242/s1, Table S1: List and accession numbers of the individuals used in all analysis; Table S2: Mitochondrial tRNA structural features of *Batagur baska* and comparative tRNA-Ser2 (trnS2) variation across *Batagur*; Table S3: Comparative start- and termination-codon patterns across seven *Batagur* mitogenomes; Table S4: Genome-wide nucleotide substitutions identified between the published *Batagur baska* reference mitogenome (BB02) and the corrected PZ210608 mitogenome; Table S5: Pairwise codon comparison between the published *B. baska* BB02 (PV156655.1) mitogenome and the corrected PZ210608 mitogenome; Table S6: Comparative analysis of codon variation across ten *B. baska* mitochondrial genomes; Table S7: Comparative analysis of amino acid variation across ten *B. baska* mitochondrial genomes; Table S8: Indel sites identified from multiple-sequence alignment of *B. baska* mitogenomes; Table S9: Synonymous and nonsynonymous substitution rates among mitochondrial protein-coding genes of seven *Batagur* species [59]; Table S10: Tandem-repeat predictions in the control regions of eight newly generated *B. baska* mitogenomes. Figure S1: Comparative mitochondrial tRNA-Ser2 (trnS2) secondary structures across 16 *Batagur* mitogenomes; Figure S2: Bayesian inference phylogram of *Batagur* mitogenomes; Figure S3: Maximum-likelihood sensitivity analysis (96-taxon dataset) excluding the eight newly generated *Batagur baska* mitogenomes, showing that major *Batagur* relationships remain unchanged in the absence of the new assemblies.
