# Supplementary material for: Elucidating the Evolutionary History of the Critically Endangered Batagur baska Using Complete Mitogenome
Source: Genes (Basel). 2026 Oct 9;17(10):1242. doi: 10.3390/genes17101242 (PMC13704110; doi:10.3390/genes17101242)
Supplement: Supplementary file 1 [file genes-17-01242-s001.zip › Supplementary Figure S1.pdf]

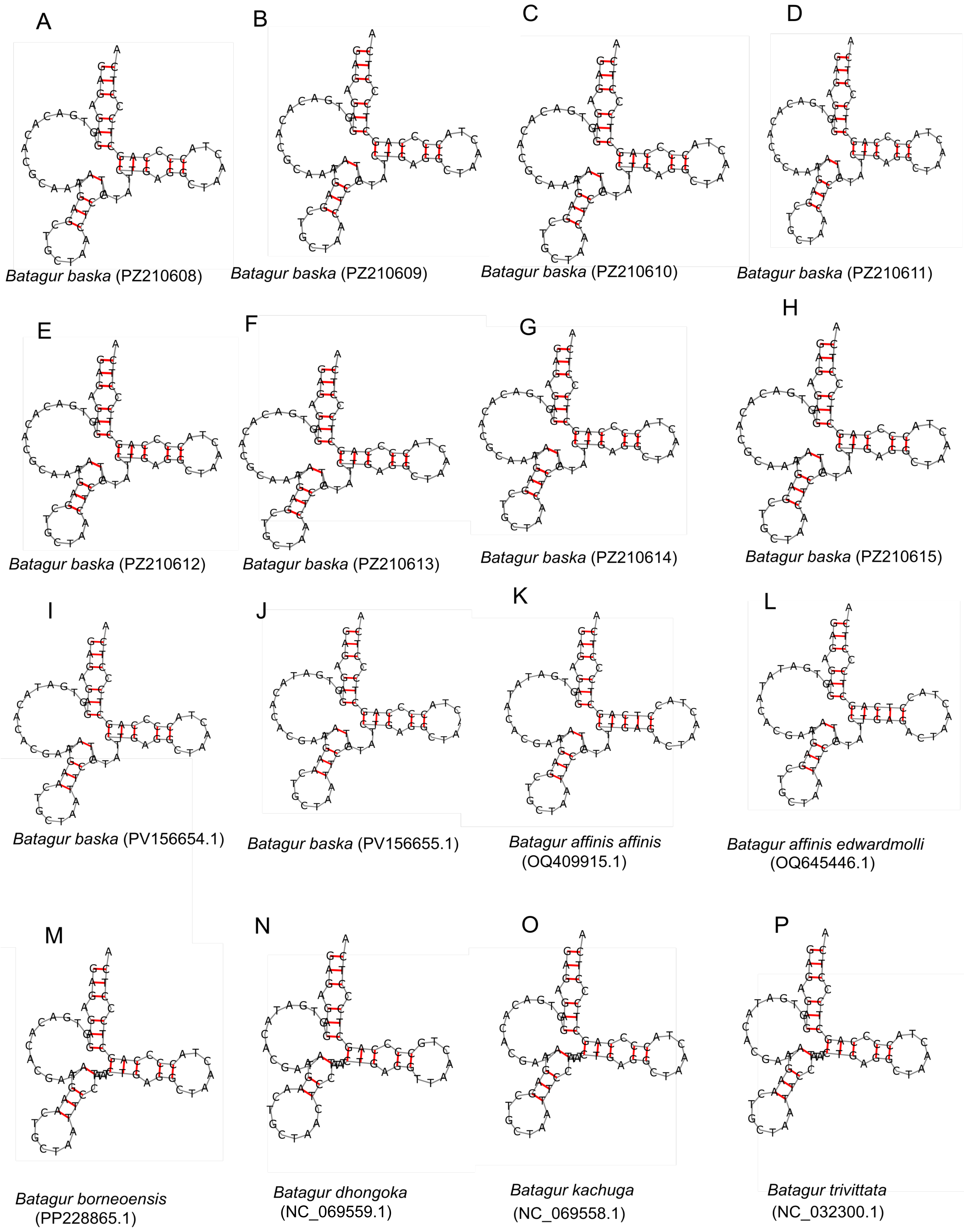

Supplementary Figure 1. Comparative mitochondrial tRNA-Ser2 (trnS2) secondary structures across 16 *Batagur* mitogenomes. Panels A–P represent the 16 mitogenomes analyzed. Footnote: Structures were predicted using RNAfold and visualized with RNAplot and are presented on a white background; species names, accession numbers, and panel letters correspond to the mitogenomes analyzed.
