# Supplementary material for: Elucidating the Evolutionary History of the Critically Endangered Batagur baska Using Complete Mitogenome
Source: Genes (Basel). 2026 Oct 9;17(10):1242. doi: 10.3390/genes17101242 (PMC13704110; doi:10.3390/genes17101242)
Supplement: Supplementary file 1 [file genes-17-01242-s001.zip › Supplementary Figure S2-Bayesian inference phylogram of Batagur mitogenomes.pdf]

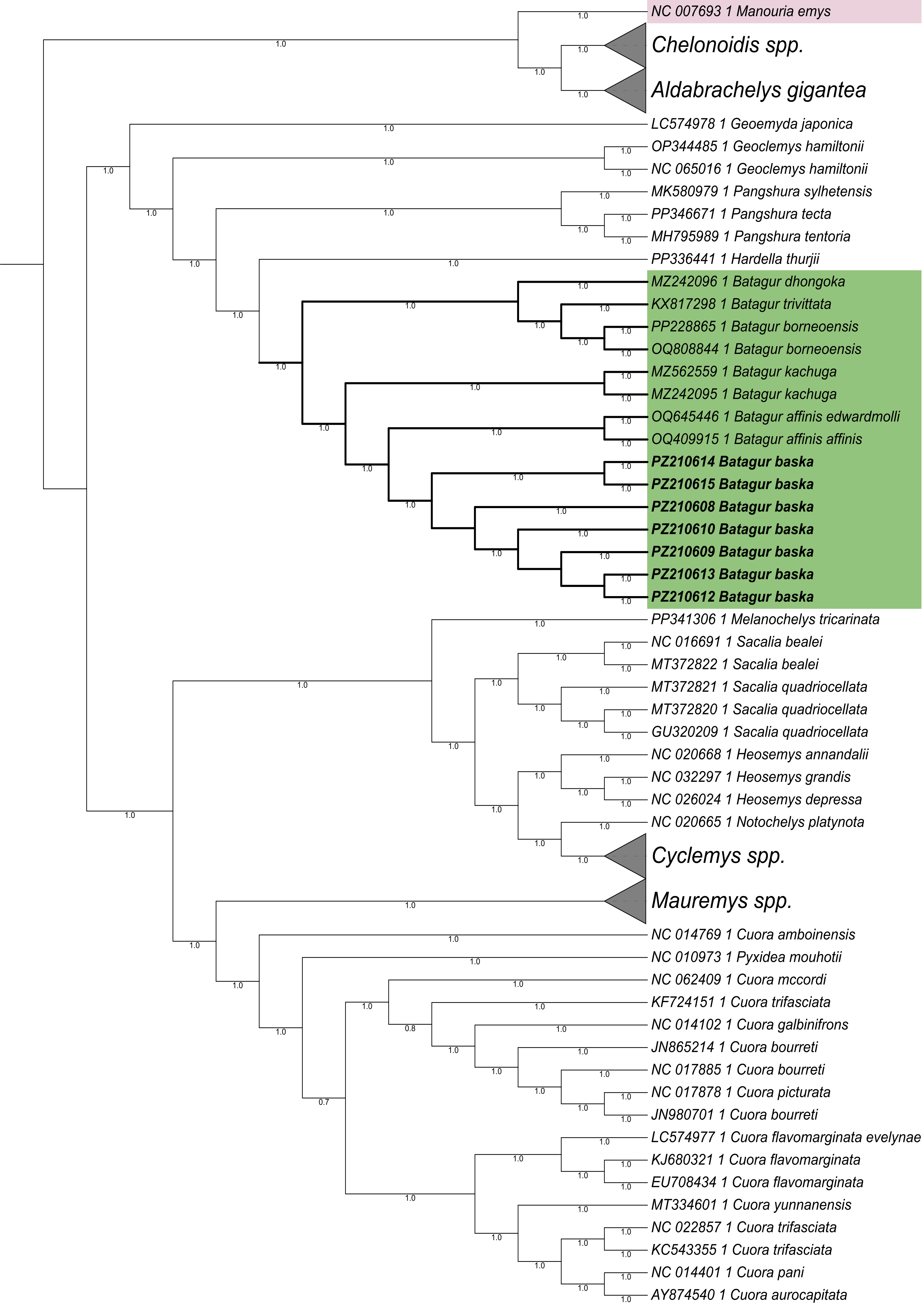
NC 007693 1 Manouria emys
Chelonoidis spp.
Aldabrachelys gigantea
LC574978 1 Geoemyda japonica
OP344485 1 Geoclemys hamiltonii
NC 065016 1 Geoclemys hamiltonii
MK580979 1 Pangshura sylhetensis
PP346671 1 Pangshura tecta
MH795989 1 Pangshura tentoria
PP336441 1 Hardella thurjii
MZ242096 1 Batagur dhongoka
KX817298 1 Batagur trivittata
PP228865 1 Batagur borneoensis
OQ808844 1 Batagur borneoensis
MZ562559 1 Batagur kachuga
MZ242095 1 Batagur kachuga
OQ645446 1 Batagur affinis edwardmolli
OQ409915 1 Batagur affinis affinis
PZ210614 Batagur baska
PZ210615 Batagur baska
PZ210608 Batagur baska
PZ210610 Batagur baska
PZ210609 Batagur baska
PZ210613 Batagur baska
PZ210612 Batagur baska
PP341306 1 Melanochelys tricarinata
NC 016691 1 Sacalia bealei
MT372822 1 Sacalia bealei
MT372821 1 Sacalia quadriocellata
MT372820 1 Sacalia quadriocellata
GU320209 1 Sacalia quadriocellata
NC 020668 1 Heosemys annandalii
NC 032297 1 Heosemys grandis
NC 026024 1 Heosemys depressa
NC 020665 1 Notochelys platynota
Cyclemys spp.
Mauremys spp.
NC 014769 1 Cuora amboinensis
NC 010973 1 Pyxidea mouhotii
NC 062409 1 Cuora mccordi
KF724151 1 Cuora trifasciata
NC 014102 1 Cuora galbinifrons
JN865214 1 Cuora bourreti
NC 017885 1 Cuora bourreti
NC 017878 1 Cuora picturata
JN980701 1 Cuora bourreti
LC574977 1 Cuora flavomarginata evelynae
KJ680321 1 Cuora flavomarginata
EU708434 1 Cuora flavomarginata
MT334601 1 Cuora yunnanensis
NC 022857 1 Cuora trifasciata
KC543355 1 Cuora trifasciata
NC 014401 1 Cuora pani
AY874540 1 Cuora aurocapitata
