# Supplementary material for: Elucidating the Evolutionary History of the Critically Endangered Batagur baska Using Complete Mitogenome
Source: Genes (Basel). 2026 Oct 9;17(10):1242. doi: 10.3390/genes17101242 (PMC13704110; doi:10.3390/genes17101242)
Supplement: Supplementary file 1 [file genes-17-01242-s001.zip › Supplementary Figure S3-Maximum-likelihood sensitivity analysis (96-taxon dataset) excluding the eight newly generated Batagur.pdf]

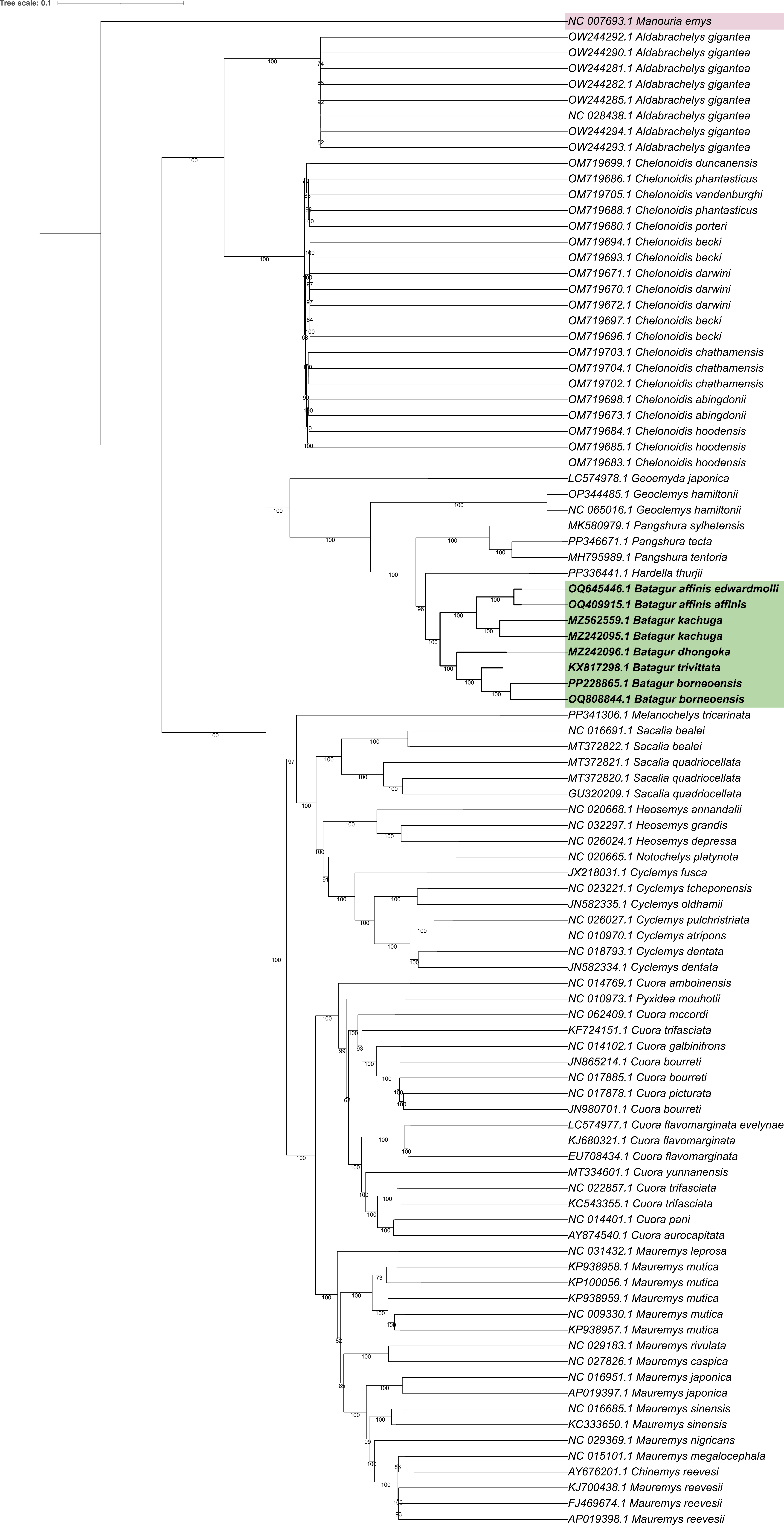
Tree scale: 0.1
NC 007693.1 Manouria emys
OW244292.1 Aldabrachelys gigantea
OW244290.1 Aldabrachelys gigantea
OW244281.1 Aldabrachelys gigantea
OW244282.1 Aldabrachelys gigantea
OW244285.1 Aldabrachelys gigantea
NC 028438.1 Aldabrachelys gigantea
OW244294.1 Aldabrachelys gigantea
OW244293.1 Aldabrachelys gigantea
OM719699.1 Chelonoidis duncanensis
OM719686.1 Chelonoidis phantasticus
OM719705.1 Chelonoidis vandenburghi
OM719688.1 Chelonoidis phantasticus
OM719680.1 Chelonoidis porteri
OM719694.1 Chelonoidis becki
OM719693.1 Chelonoidis becki
OM719671.1 Chelonoidis darwini
OM719670.1 Chelonoidis darwini
OM719672.1 Chelonoidis darwini
OM719697.1 Chelonoidis becki
OM719696.1 Chelonoidis becki
OM719703.1 Chelonoidis chathamensis
OM719704.1 Chelonoidis chathamensis
OM719702.1 Chelonoidis chathamensis
OM719698.1 Chelonoidis abingdonii
OM719673.1 Chelonoidis abingdonii
OM719684.1 Chelonoidis hoodensis
OM719685.1 Chelonoidis hoodensis
OM719683.1 Chelonoidis hoodensis
LC574978.1 Geoemyda japonica
OP344485.1 Geoclemys hamiltonii
NC 065016.1 Geoclemys hamiltonii
MK580979.1 Pangshura sylhetensis
PP346671.1 Pangshura tecta
MH795989.1 Pangshura tentoria
PP336441.1 Hardella thurjii
OQ645446.1 Batagur affinis edwardmolli
OQ409915.1 Batagur affinis affinis
MZ562559.1 Batagur kachuga
MZ242095.1 Batagur kachuga
MZ242096.1 Batagur dhongoka
KX817298.1 Batagur trivittata
PP228865.1 Batagur borneoensis
OQ808844.1 Batagur borneoensis
PP341306.1 Melanochelys tricarinata
NC 016691.1 Sacalia bealei
MT372822.1 Sacalia bealei
MT372821.1 Sacalia quadriocellata
MT372820.1 Sacalia quadriocellata
GU320209.1 Sacalia quadriocellata
NC 020668.1 Heosemys annandalii
NC 032297.1 Heosemys grandis
NC 026024.1 Heosemys depressa
NC 020665.1 Notochelys platynota
JX218031.1 Cyclemys fusca
NC 023221.1 Cyclemys tcheponensis
JN582335.1 Cyclemys oldhamii
NC 026027.1 Cyclemys pulchristriata
NC 010970.1 Cyclemys atripons
NC 018793.1 Cyclemys dentata
JN582334.1 Cyclemys dentata
NC 014769.1 Cuora amboinensis
NC 010973.1 Pyxidea mouhotii
NC 062409.1 Cuora mccordi
KF724151.1 Cuora trifasciata
NC 014102.1 Cuora galbinifrons
JN865214.1 Cuora bourreti
NC 017885.1 Cuora bourreti
NC 017878.1 Cuora picturata
JN980701.1 Cuora bourreti
LC574977.1 Cuora flavomarginata evelynae
KJ680321.1 Cuora flavomarginata
EU708434.1 Cuora flavomarginata
MT334601.1 Cuora yunnanensis
NC 022857.1 Cuora trifasciata
KC543355.1 Cuora trifasciata
NC 014401.1 Cuora pani
AY874540.1 Cuora aurocapitata
NC 031432.1 Mauremys leprosa
KP938958.1 Mauremys mutica
KP100056.1 Mauremys mutica
KP938959.1 Mauremys mutica
NC 009330.1 Mauremys mutica
KP938957.1 Mauremys mutica
NC 029183.1 Mauremys rivulata
NC 027826.1 Mauremys caspica
NC 016951.1 Mauremys japonica
AP019397.1 Mauremys japonica
NC 016685.1 Mauremys sinensis
KC333650.1 Mauremys sinensis
NC 029369.1 Mauremys nigricans
NC 015101.1 Mauremys megalocephala
AY676201.1 Chinemys reevesi
KJ700438.1 Mauremys reevesii
FJ469674.1 Mauremys reevesii
AP019398.1 Mauremys reevesii
