# Supplementary material for: Elucidating the Evolutionary History of the Critically Endangered Batagur baska Using Complete Mitogenome
Source: Genes (Basel). 2026 Oct 9;17(10):1242. doi: 10.3390/genes17101242 (PMC13704110; doi:10.3390/genes17101242)
Supplement: Supplementary file 1 [file genes-17-01242-s001.zip › Supplementary Table S9.pdf]

Supplementary Table S9: Synonymous and nonsynonymous substitution rates among mitochondrial protein-coding genes of seven *Batagur* species.

PCG	Mean dN	Mean dS	dN/dS (ω)	Pairwise ω range
ATP8	0.0767	0.2091	**0.3667**	0.2207–1.1546
ATP6	0.0272	0.3568	**0.0763**	0.0458–0.1894
COX1	0.0037	0.2320	**0.0161**	0.0000–0.0524
COX2	0.0089	0.2342	**0.0379**	0.0000–0.0831
COX3	0.0058	0.2397	**0.0241**	0.0000–0.0441
ND1	0.0260	0.3255	**0.0800**	0.0619–0.1820
ND2	0.0306	0.3700	**0.0826**	0.0353–0.1266
ND3	0.0463	0.2162	**0.2143**	0.1076–0.8603
ND4L	0.0321	0.2496	**0.1286**	0.0000–0.3833
ND4	0.0261	0.3044	**0.0859**	0.0606–0.1567
ND5	0.0274	0.3318	**0.0825**	0.0463–0.4283
ND6	0.0614	0.2248	**0.2730**	0.0446–0.5174
CYTB	0.0143	0.3914	**0.0366**	0.0204–0.0632

Note. Mean dN and dS represent the average of 21 pairwise Yang–Nielsen (YN00) estimates among seven *Batagur* taxa for each PCG. The reported dN/dS (ω) was calculated as mean dN divided by mean dS. Pairwise ω range represents the minimum and maximum YN00 estimates among the 21 pairwise comparisons.

1. **Methodological reference: 59:**

2. Yang, Z.; Nielsen, R. Estimating synonymous and nonsynonymous substitution rates under realistic evolutionary models. *Mol. Biol. Evol.* **2000**, *17*, 32–43. https://doi.org/10.1093/oxfordjournals.molbev.a026236

Analysis note: The table reports descriptive pairwise substitution-rate estimates and gene-wise ω values. The isolated ATP8 pairwise estimate above 1 should not be interpreted alone as evidence of positive selection.
